# A simple knowledge-based mining method for exploring hidden key molecules in a human biomolecular network

**DOI:** 10.1186/1752-0509-6-124

**Published:** 2012-09-15

**Authors:** Shingo Tsuji, Sigeo Ihara, Hiroyuki Aburatani

**Affiliations:** 1 Genome Science Division, Research Center for Advanced Science and Technology (RCAST), The University of Tokyo, 4-6-1 Komaba, Meguro-ku, Tokyo 153-8904, Japan; 2 Komaba Open Laboratory, The University of Tokyo, Tokyo, Japan

**Keywords:** Knowledge-based analysis, Network data mining, Omics data analysis, Cancer research

## Abstract

**Background:**

In the functional genomics analysis domain, various methodologies are available for interpreting the results produced by high-throughput biological experiments. These methods commonly use a list of genes as an analysis input, and most of them produce a more complicated list of genes or pathways as the results of the analysis. Although there are several network-based methods, which detect key nodes in the network, the results tend to include well-studied, major hub genes.

**Results:**

To mine the molecules that have biological meaning but to fewer degrees than major hubs, we propose, in this study, a new network-based method for selecting these hidden key molecules based on virtual information flows circulating among the input list of genes. The human biomolecular network was constructed from the Pathway Commons database, and a calculation method based on betweenness centrality was newly developed. We validated the method with the ErbB pathway and applied it to practical cancer research data. We were able to confirm that the output genes, despite having fewer edges than major hubs, have biological meanings that were able to be invoked by the input list of genes.

**Conclusions:**

The developed method, named NetHiKe (Network-based Hidden Key molecule miner), was able to detect potential key molecules by utilizing the human biomolecular network as a knowledge base. Thus, it is hoped that this method will enhance the progress of biological data analysis in the whole-genome research era.

## Background

The emergence of next-generation sequencing technology and sophisticated microarray technology has enhanced the diversity of high-throughput biological experiments. In addition to gene expression profiling, epigenetic data, including DNA methylation and histone modifications, and mutation analysis in cancer have been studied comprehensively in a genome-wide manner. It is absolutely indispensable to use biological knowledge-based analysis methods to translate the results of these experiments into a better understanding of the underlying phenomena and to plan the next stages of research.

Biological knowledge, such as pathways or gene sets, is compiled in various databases. In these databases, biological knowledge is represented as a precompiled, divided set of genes, such as the “P53 signaling pathway” or “apoptotic signaling pathway”. These pathways are utilized by various knowledge-based analysis methods. Over-representation analysis (ORA) is a widely used method for mapping a list of genes onto these pathways automatically, and this technique can determine the pathways or functional gene sets that are enriched in a given list of genes obtained experimentally. ORA is frequently implemented as a web application, such as the NCI-Nature Pathway Interaction Database [1,2] and the DAVID bioinformatics resources [3], that receive an input list of genes and calculate the p-values based on the frequency of the appearance of the input genes in each precompiled gene set. However, using the ORA methodology, the input list of genes is simply characterized with respect to the already-known pathways. Thus, researchers can rarely discover something new related to their input.

Another type of knowledge-based analysis is the network-based analysis method, which uses an interaction network of biomolecules as the knowledge. In this type of network, the biomolecules (proteins or genes) correspond to the nodes, and the edges indicate the relationships between the molecules (e.g., “protein A induces protein B” or “protein B phosphorylates protein C”). The assembled network is often called a protein-protein interaction (PPI) network or a biomolecular network, and several methodologies are available for analyzing experimental results using this network-based biological knowledge [4-6]. Many network-based analysis methods extract modules, which are sets of tightly connected nodes consisting of the input genes, and it is strongly expected that the genes in a module achieve a biological function in a coordinated manner. In addition, these modules sometimes include nodes that were not present in the input list. Thus, the network-based analysis methods partially overcome the disadvantages of ORA, in terms of the limitation to the predefined pathways or gene sets. However, these module-centric methods restrict the results of the analysis to a certain area of each module, even though the input genes are spread over the whole biomolecular network. Furthermore, when the modules of the analysis results become larger or more complex, it is almost impossible to understand their biological meanings.

Consequently, it would be beneficial to identify the nodes in the network as the key molecules that are relevant to the input list of genes. One of the most prominent characteristics of a node in a network is its degree, or number of neighbors. However, the degree contains information only about its neighbors, and in a similar way, other network measures, such as the clustering coefficient and assortativity, merely reflect the situations of their neighbors [7]. In contrast, certain node centralities can determine the importance of each node in a network by taking into consideration the topology of the entire network. Although there are various types of centralities, such as degree centrality, closeness centrality, eigenvector centrality, betweenness centrality and others, it is known that almost all of the centralities correlate with the degree of the node [8]. Partially because the role of hub nodes in biomolecular networks still remains an intensive research target [9-11], the methods based on these centralities [12-14] tend to produce analysis results that are biased toward major hub nodes.

In this study, we present a new network-based method for identifying the hidden key molecules, a description that indicates that the molecules are biologically relevant to the input but do not have as many neighbors as the major hub nodes have. We have developed a centrality measure derived from betweenness centrality [15,16], named node-limited betweenness centrality (nlBC). First, we validated the method using a well-known pathway, the ErbB (EGFR) signaling pathway. Next, we applied it to a practical cancer mutation dataset and explored the availability of our method.

## Results and discussion

### Methodology overview

**Figure 1:**
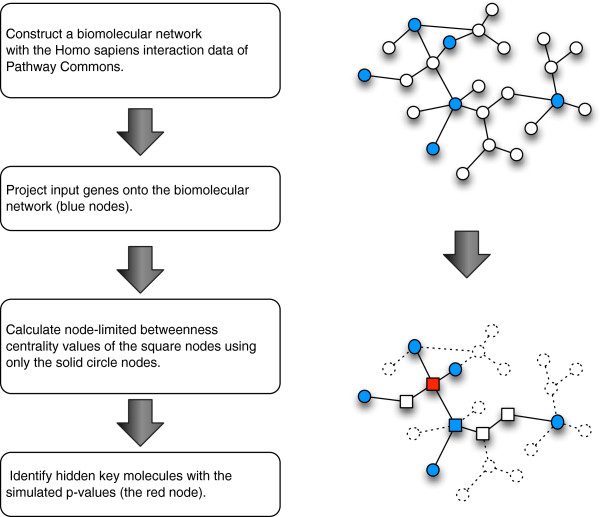
**Schematic view of the NetHiKe method.** A schematic view of Network-based Hidden Key Molecule Miner (NetHiKe). The blue nodes indicate the input nodes, and only the square nodes have node-limited betweenness centrality values. The red node indicates the statistical significance by the simulated p-values.

Figure 1 shows a schematic view of our method. We call this method Network-based Hidden Key Molecule Miner (NetHiKe), and a detailed description is provided in the “Methods” section. First, we constructed a biomolecular network as an undirected graph, which represents the knowledge about the interactions among the biomolecules (genes or proteins) using Pathway Commons data. Then, we projected the input genes onto the network and calculated the newly developed centrality values of the nodes. To calculate the centrality, we used only the shortest paths that have both ends in the set of the input nodes. Thus, the only nodes that were included in the network consisted of the shortest paths between all the possible combinations of any two input nodes with the centrality values. We named this centrality value the “node-limited betweenness centrality (nlBC)”, and this method can utilize the sum of the weight values of both ends of each shortest path (see “Methods” for details). The significance of the nlBC was assessed by p-values based on a Monte-Carlo simulation, by generating the same number of randomly selected nodes as the input nodes.

### Verification of the Method

First, we conducted the following computational experiments to verify whether the developed method has the ability to extract the appropriate knowledge related to the input data. As with the input data, we used a gene list that consisted of 10 ligands and 30 transcription factors of the ErbB pathway (listed in Additional file 1, and see “Methods” for details). The results of this analysis are listed in Table 1. This table contains the list of 31 genes whose simulated p-values were under 0.05, in ascending order of the p-values. The list also indicates the degree of the node in the background network, nlBC, and whether the gene was included in the input list.

**Table 1:** The results of the ErbB pathway analysis

**Gene symbol**	**Degree**	**nlBC**	**Included in** **the input**	**Simulated** **p-value**
EGFR	129	0.248	no	5*.*01×10^−5^
JUN	90	0.0854	yes	1*.*17×10^−3^
CREBBP	124	0.0919	no	1*.*42×10^−3^
TCF3	24	0.0431	no	1*.*59×10^−3^
FOXO4	9	0.00429	no	2*.*62×10^−3^
EP300	146	0.102	no	2*.*70×10^−3^
ERBB2	33	0.0274	no	6*.*05×10^−3^
CDC25A	18	0.0149	no	8*.*28×10^−3^
CABIN1	8	0.0369	no	9*.*42×10^−3^
ERBB3	13	0.0329	no	9*.*76×10^−3^
TFDP2	4	0.0039	no	1*.*12×10^−2^
CEBPB	34	0.0261	no	1*.*18×10^−2^
BAG1	13	0.00881	no	1*.*19×10^−2^
ID2	17	0.0318	no	1*.*28×10^−2^
MEF2D	11	0.00573	yes	1*.*34×10^−2^
MYBL2	13	0.0445	no	1*.*44×10^−2^
ERBB4	19	0.0304	no	1*.*74×10^−2^
SP1	59	0.0288	no	1*.*92×10^−2^
RB1	92	0.061	no	2*.*02×10^−2^
HCFC1	26	0.0352	no	2*.*11×10^−2^
RYBP	13	0.0155	no	2*.*12×10^−2^
E2F4	21	0.00537	yes	2*.*31×10^−2^
USP7	42	0.0606	no	2*.*41×10^−2^
SRF	26	0.0427	no	2*.*85×10^−2^
TFDP1	14	0.00927	no	2*.*96×10^−2^
RBL2	25	0.00674	no	3*.*01×10^−2^
STAT1	50	0.026	yes	3*.*06×10^−2^
E2F1	45	0.0282	yes	3*.*31×10^−2^
ATF2	27	0.00428	no	4*.*78×10^−2^
CEBPA	21	0.00393	no	4*.*89×10^−2^
YWHAQ	63	0.0189	no	4*.*93×10^−2^

Key molecules (genes) with simulated p-values less than 0.05 are listed in the order of ascending p-values. The degree is the number of edges (number of interaction partners), and the fourth column indicates whether the gene was included in the input list of genes.

**Figure 2:**
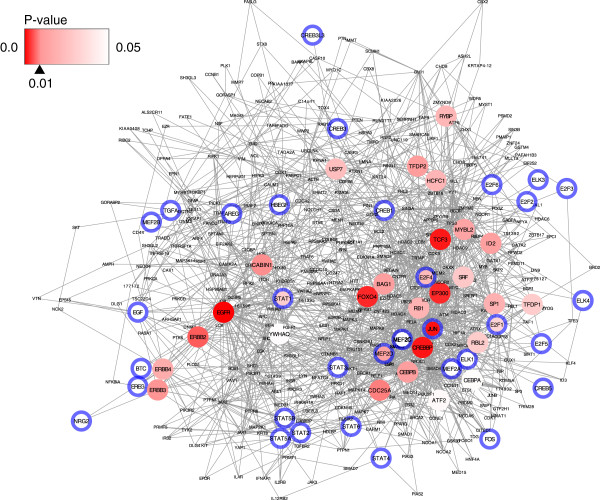
**Network visualization of the results of the ErbB pathway analysis.** The extracted subgraph (420 nodes and 1,575 edges) induced by the input genes. The red color depth represents the p-values, which were estimated by the simulations, and the blue boundaries indicate that the node was included in the input list.

The output list includes all four transmembrane tyrosine kinase receptors: the epidermal growth factor receptor (EGFR; also known as ERBB1), ERBB2, ERBB3 and ERBB4. These four receptor genes were not included in the input; NetHiKe successfully detected these four key molecules, which were deeply relevant to the 10 ligands in the input list. The transcription factors of the ErbB pathway, such as Jun, E2F, STAT and MEF2, are presented in Table 1, and these factors were included in the input list. This observation means that NetHiKe can mark a molecule as key even when the node is in the input list. The network view of this result is shown in Figure 2. This figure contains all of the pairs of the shortest paths among the inputs. In this figure, we can verify that NetHiKe appropriately detected ErbB pathway related genes, such as FOXO4 and CREBBP.

To confirm the biological meanings of the results, we analyzed the genes in Table 1 using the Pathway Interaction Database, which is one of the typical over-representation analysis methods (see “Methods” for details). As shown in Additional file 2A (the link to NetHiKe), we obtained “E2F transcription factor network” as the most significant pathway, which is one of the downstream effects of an ErbB pathway stimulus.

#### The relationship between nlBC and P-values

To illustrate the properties of the nlBC and its p-values, we constructed individuals scatter plots for the nlBC, degree and p-value for the genes listed in Table 1 (Additional files 3A to 3C). The nlBC values modestly correlate with degree (Additional file 3A), whereas the p-value has almost no relationship with degree or nlBC (Additional files 3B and 3C). To understand the behavior of nlBC and its p-value and to determine the robustness of nlBC, we constructed a boxplot to visualize the nlBC values for the genes in Table 1 (Additional file 3D and 3E). In the plots, the boxes of Additional file 3D show the nlBCs that were generated using randomly selected genes for calculating simulated p-values, and the vertical spread of the boxes are indicative of the variation of the nlBC in response to the various input list of genes. The boxes of Additional file 3E were generated by a leave-one-out method using the ErbB input genes, and the boxes are indicative of the robustness of the nlBC for certain input genes. The nlBC values vary in the different input list and their ranges also differ from each other. It seems that the ranges depend on the degree of each gene. However, the nlBC values of a certain semantic group of genes, such as those in the ErbB pathway, are significantly different from their randomly generated background distributions. Furthermore, the values are robust. Thus, to identify these alterations in the nlBC using NetHiKe, we validated the importance of the genes using simulated p-values instead of the nlBC values themselves.

#### Comparison with the Hubba results

**Figure 3:**
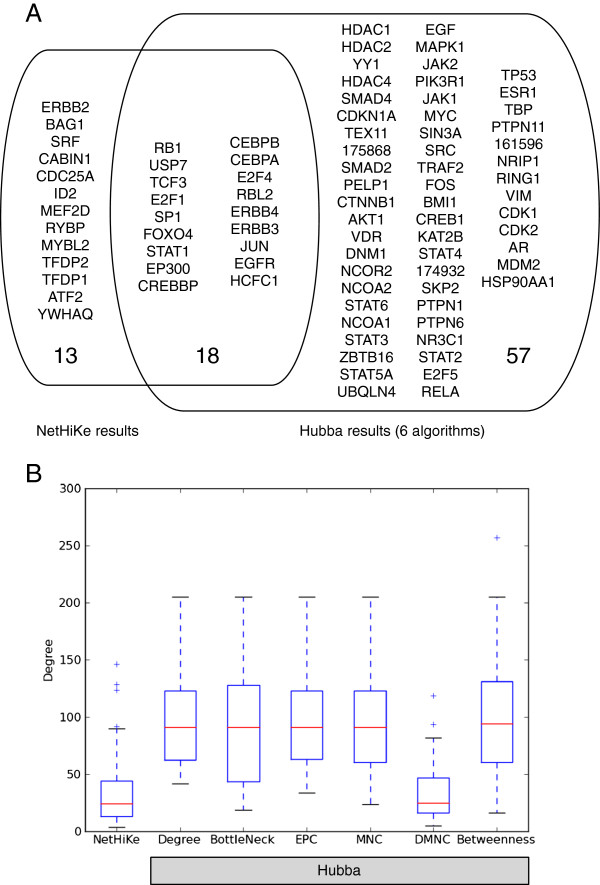
**Comparison of the results of NetHiKe and Hubba.A**) Venn diagram for the comparison of the results of NetHiKe and Hubba. The Hubba result is the combination of the six different methods. **B**) The boxplot of the degree of each node. The degree was calculated in the background network rather than in the extracted network.

To clarify the characteristics of our methods, we compared our results with the existing method. As a comparison method, we chose Hubba [12] (see “Methods” for details). We compared the top 30 genes from the NetHiKe results, chosen based on their p-values, and the Hubba results, which were produced by the six different algorithms that are implemented in Hubba. Figure 3A shows a Venn diagram of this comparison. The Hubba results have more genes than the NetHiKe results. This discrepancy occurs because the Hubba results consist of a union of the six different outputs (all of the genes are listed in Additional file 4). As shown in Additional files 2B to 2F, the Hubba results from the six different methods include ErbB pathway-related genes, such as “Glucocorticoid receptor regulatory network” and “Regulation of nuclear SMAD2/3 signaling”. This observation means that the results of Hubba also have an important role in the analysis of the ErbB pathway.

When drawing the boxplots for the degrees of the genes (Figure 3B), the degree distribution of the NetHiKe results was much smaller than that of the Hubba results excluding DMNC, one of the algorithms of Hubba. For example, EGFR (ERBB1), ERBB2, ERBB3, and ERBB4, which are four membrane receptors of the ErbB pathway, have 129, 33, 13, and 19 neighbors, respectively, in the background knowledge-base network. EGFR is considered to be one of the major hubs in this network, and Hubba (DMNC), whose degree distribution was as small as that of NetHiKe, failed to detect EGFR. In contrast, only the NetHiKe result has all four of these receptors in the top 30 gene list. Recently, ERBB2 and ERBB3, which have fewer degrees than EGFR, have been considered to play key roles in cancer tissue [17,18]. These results suggest that NetHiKe can detect the hidden key molecules based on the context in which an input list of genes is given.

#### Weighted inputs

**Figure 4:**
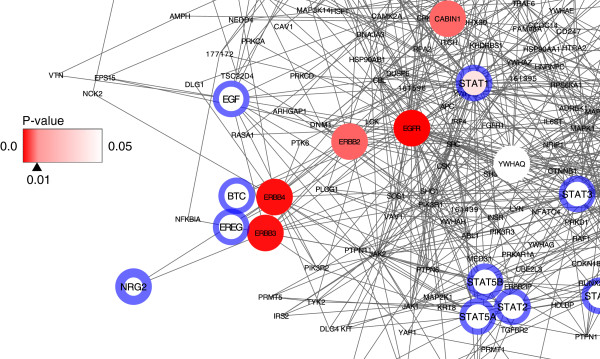
**Network visualization with weighted input.** Network visualization for the case of the weighted input, in which only NRG2 has double weight relative to the others. The figure shows only the area around NRG2 and its receptors (ERBB3 and ERBB4). As NRG2 is one of the input genes, it has a blue line around the node, and its greater width indicates a greater weighted value relative to the other input genes.

Finally, we have validated the function for handling the weighted values of the input nodes. The weight of NRG2, which is one of the input genes in this validation study, was set to 2.0, and the remainder of the input nodes had their weight values set to 1.0. The results of the analysis are shown in table format in Additional file 5. Overall, there were many overlaps between the NRG2 weighted results and the non-weighted results, such as JUN, TCF3, CREBBP and EP300 (Additional file 5A and Table 1). This observation could also be confirmed by the results of an analysis using the Pathway Interaction Database (Additional files 2A and 2G). The network visualization near ERBB receptor family is shown in Figure 4. We can confirm that the red color of ERBB3 and ERBB4 is deeper than that of Figure 1, which was produced using the non-weighted input list. This finding means that the p-values of these genes became more significant, with p-values less than 0.05 to 0.01, and the results were satisfactory considering that NRG2 is the ligand for ERBB3 and ERBB4, not for ERBB1 and ERBB2 [19].

When the weight value of NRG2 was increased to 20.0, the results included more ERBB4-related genes (the result table is shown in Additional file 5). To confirm this finding, we again examined the results using the Pathway Interaction Database. As shown in Additional file 2H, “ERBB4 signaling events” was the second most important pathway because the increased weight of NRG2, the ligand of ERBB4, appropriately enhances the importance of ERBB4-related pathways. Taking these results together, if appropriate weights are given to NetHiKe, this algorithm can detect the nodes that have biological meaning but do not have many edges with statistical significance, such as *p*<0*.*01.

### Analysis of practical cancer mutation data

We applied the NetHiKe algorithm to the list of genes that are somatically mutated in glioblastomas using their observed mutation frequencies as the weight values. The input mutation data were obtained from The Cancer Genome Atlas (TCGA) website (see the “Methods” for details about TCGA and glioblastoma). The analysis results are shown in Table 2 and Additional file 6. The genes with p-values less than 0.01 are listed in Table 2, and the whole network is visualized in Additional file 6. In Additional file 6, PTEN, TP53 and EGFR have a thicker blue border than the other genes because they have an extremely high mutation rate in glioblastomas. (See Additional file 1 for the numerical data).

**Table 2:** The results of GBM mutation data

**Gene symbol**	**Degree**	**nlBC**	**Included in the input**	**Simulated p-value**
PTK2	20	0.014	no	5*.*02×10^−5^
BCAR1	17	0.00496	yes	1*.*52×10^−3^
CD36	11	0.00336	no	2*.*15×10^−3^
PIK3CB	10	0.00525	no	2*.*70×10^−3^
MAP2	6	0.00223	no	3*.*81×10^−3^
DHFR	4	0.00318	no	4*.*62×10^−3^
VAV3	11	0.00483	no	5*.*44×10^−3^
ITGB3	37	0.0273	yes	5*.*83×10^−3^
SP3	9	0.00678	no	6*.*44×10^−3^
TXN	8	0.00692	no	6*.*83×10^−3^
DAP3	8	0.00269	no	7*.*22×10^−3^
RUNX2	22	0.0111	no	7*.*78×10^−3^
NR2F1	8	0.0239	no	8*.*61×10^−3^
PXN	45	0.0198	no	8*.*85×10^−3^
RPS27L	9	0.00121	no	9*.*09×10^−3^
PTPN1	33	0.018	no	9*.*50×10^−3^

The table shows the key molecules (*p*<0*.*01) that were inferred by NetHiKe, with their degrees and nlBC values. The genes are ordered by their simulated p-values.

As shown in Table 2, the NetHiKe results do not include several famous key players in glioblastoma biology, such as EGFR, SRC and TP53 [20]. However, the nodes with fewer edges than those above that are included also have implications in glioblastoma biology. PTK2 (also known as FAK: focal-adhesion kinase), which is the top-ranked gene in Table 2, is a non-receptor tyrosine kinase protein that serves as a major mediator of cell migration [21], and the suppression of PTK2 phosphorylation inhibits glioma cell migration [22]. PTK2 is also gaining attention as a drug target in cancer therapy; for example, a kinase inhibitor of PTK2 has been developed in ovarian cancer [23]. Clinical studies on pancreatic cancer [24] and neuroblastoma [25], which is the most common childhood brain cancer, are also under way. PXN (also known as Paxillin), which is one of the hidden key molecules (Table 2), is known to be a downstream target of PTK2. Additionally, the PTK2(FAK)-signaling pathway, which is formed by these genes, has been shown to be upstream of AKT-signaling in promoting malignant behaviors of high-grade gliomas [26]. BCAR1 (also known as p130Cas), which is the second most significant key molecule, is also known to be a mediator of growth factor-dependent migration through tyrosine phosphorylation in glioma cells [27].

**Figure 5:**
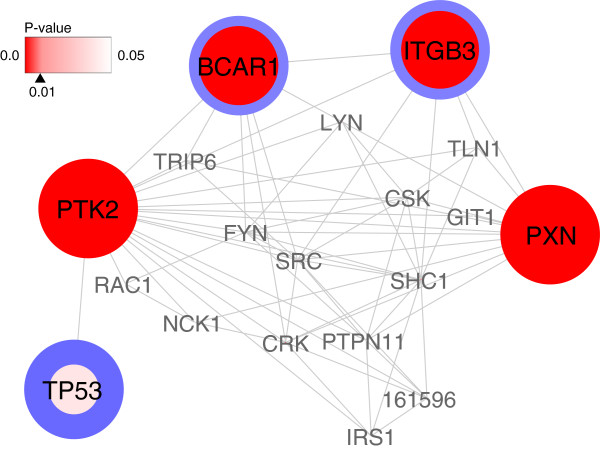
**PTK2 and its neighbors.** The network visualization of PTK2 and its neighbor nodes with input weight values (thickness of blue border line) and P-values (depth of red color). PTK2 has a direct relationship with BCAR1 and PXN, as well as TP53, which is well-known to be one of the most famous hub genes.

Figure 5 shows the neighbor nodes of PTK2, which were extracted and visualized by Cytoscape, and this figure shows that PTK2 associates not only with PXN but also with BCAR1. Although the role of the relationship between PTK2 and ITGB3 in glioma biology is not clear, ITGB3 (integrin *β*3) plays a pro-apoptotic role in glioma cells, and it is related to anti-cancer drug resistance [28]. These results suggest that NetHiKe can detect the molecules that are deeply related to the biological background of the brain tumor.

#### Comparison to Hubba

We compared the NetHiKe results with the Hubba results as an existing similar method. Because Hubba cannot manipulate the node weights, we used only the gene names as an input for Hubba with the six different algorithms, as in the ERBB comparison case (see the “Methods” for the details). Additional file 7 shows the top 16 genes of the six Hubba methods, which represents the same number of genes found in the NetHiKe results with *p*<0*.*01. There were no overlapping genes between the NetHiKe results and the Hubba results. In contrast, there were several overlapping genes among the six Hubba methods. When we mapped the differentially expressed genes in glioblastoma obtained from TCGA to these results, the genes were distributed across all of the results from both NetHiKe and Hubba. This observation could indicate that the listed genes of both methods are related to glioblastoma biology. For example, MAP2, which was selected by NetHiKe and is differentially expressed in glioma, is known to be one of the neuronal differentiation markers, and its expression level is naturally decreased in brain tumors [29].

Table 3 shows the genes that were presented at least three times in all six methods of generating Hubba results. Obviously, the genes selected by Hubba have much greater degrees than the genes selected by NetHiKe (Additional file 7, also Tables 2 and 3). Many of the genes selected by Hubba are known to be major key players in glioma biology, such as EGFR, EP300, SRC and TP53 [20]. Although the NetHiKe results tend to have fewer degrees, they have relationships to these major genes to a certain extent. For instance, SRC and TP53, which were selected in the Hubba results many times, are presented in Figure 5 as the neighbors of PTK2. RAC1 and SHC1, which were also selected by Hubba (Additional file 7), associate with PTK2 (Figure 5), and the association between SHC1 and PTK2 has been known for a long time [30].

**Table 3:** The summary of the Hubba results for GBM data

**Gene**	**Degree**
EGFR	129
EP300	146
HSP90AA1	166
HSPA8	111
PRKDC	112
SHC1	110
SRC	134
TP53	132
YWHAG	183

The genes that were represented at least three times among the results of the six different Hubba algorithms.

Therefore, these results show that NetHiKe captures the nodes that are on the periphery of the major hub nodes. We think that this outcome arises because nlBC includes only the shortest paths with both ends in the input nodes. This characteristic reduces the shortest paths that are concentrated on the major hubs with no relationships to the input genes. Consequently, NetHiKe is able to mine the hidden key molecules that have sufficient biological meaning and fewer degrees than the major hub nodes.

## Conclusions

We have proposed an analysis method, Network-based Hidden Key Molecule Miner (NetHiKe), which can extract limited numbers of hidden key molecules relevant to genes provided as input, using a human biomolecular network. NetHiKe comprises three steps: mapping the input genes onto the network, a node-limited betweenness centrality (nlBC) calculation, and validation of the statistical significance by simulated p-values. NetHiKe tends to capture the nodes with fewer degrees than major hub nodes, which are usually intensive research targets. We have confirmed that NetHiKe’s outputs contain sufficient biological information and that the input node weights appropriately produce a change in the results based on the biological meanings. Furthermore, with the glioblastoma analysis, we demonstrated that NetHiKe can be used for analyzing practical biology data produced by genome-wide experimental methodologies.

The present knowledge about cell biology is enormous, and thus, the derivation of informative meaning from genome-wide experimental results is urgently needed. We anticipate that this simplicity will contribute to additional striking insights into cellular activity and help researchers to determine future research directions.

## Methods

### Biomolecular network

We used the Pathway Commons [31] dataset, released on Oct 27, 2011, to construct a human biomolecular network. Pathway Commons currently includes the following nine data sources: BioGRID [32], The Cancer Cell Map [33], the HPRD [34], HumanCyc [35], the databases of the Systems Biology Center NewYork [36], IntAct [37], the Molecular Interaction Database (MINT) [38], the NCI-Nature Pathway Interaction Database [1] and Reactome [39]; thus, it includes many types of biomolecular interactions, such as biochemical reactions, complex assembly, transport and catalysis events, and physical interactions involving proteins, DNA, RNA, small molecules and complexes.

We visualized the degree distribution of the network that was constructed from the pathway commons data (Additional file 8A), and we found that there were extra high-degree nodes, which disturb the power-law of the log-log degree distribution. To obtain a more reliable biomolecular network, we extracted the binary relationships of biomolecules that represented at least two of the nine data sources used by the Pathway Commons. Again, we visualized the degree distribution of this edge-selected network; the distribution now followed the power-law clearly (Additional file 8B). We used this network in further analyses.

In a network construction step, redundant edges and self-directed edges may exist if multiple data sources include the same interaction or a multimeric protein complex. Because the nlBC algorithm described below does not take into account multiple edges or self-directed edges, all of the redundant edges were collapsed into single edges, and all of the self-directed edges were pruned from the network. Consequently, by ignoring the tiny disconnected components, we obtained a human biomolecular network: a connected, unweighted, undirected graph with 7,456 nodes and 35,553 edges.

### Node-limited betweenness centrality

A biomolecular network can be described as a graph *G *= (*V*,*E*), where the set *V * of nodes represents proteins or genes, and the set *E* of edges represents the relationships among these biomolecules. Let *σ*_*st*_ denote the number of shortest paths from the node *s*∈*V*to the node *t*∈*V*, and let *σ*_*st*_(*v*) denote the number of shortest paths from *s* to *t* that include *v*. The betweenness centrality of node *v* is determined as follows:

$$\text{BC}(v)=\underset{s\neq v\neq t\in V}{\sum }\frac{\sigma_{\text{st}}(v)}{\sigma_{\text{st}}}.$$

The betweenness centrality of a node can be calculated by counting the number of shortest paths passing through the node and the entire number of shortest paths between arbitrary pairs of nodes in the graph.

Normally, the betweenness centrality of a node is calculated based on all of the nodes in the graph. However, in this study, as we wanted to identify the nodes that have a close relationship to the input nodes, we developed a novel variant of betweenness centrality, named “node-limited betweenness centrality,” to mine the hidden key molecules from among the whole background network. The variant method includes only the shortest paths for which both ends are in the input nodes. In addition, the method can manipulate the weights of both ends.

Let *U* be the set of the input nodes; then, we can define the subgraph *H *= (*V*_*H*_,*E*_*H*_) as follows:

$$H=\underset{s\neq t\in U}{⋃}{\text{SP}}_{<s,t>}.$$

*SP*_<*s*,*t*>_denotes a path set of all the possible shortest paths from node *s* to node *t*. Node-limited betweenness centrality (nlBC) can have non-zero values when the node *v* satisfies the condition *v*∈*V*_*H*_, and the definition of this term is as follows:

$$\text{nlBC}(v)=\frac{1}{w}\underset{s\neq t\in U}{\sum }(w(s)+w(t))\frac{\sigma_{\mathrm{st}}(v)}{\sigma_{\mathrm{st}}}.$$

$$w=\underset{s\neq t\in U}{\sum }w(s)+w(t)$$

*w(x)* is the weight value of the node *x*. Under the definition of nlBC, we can define the subgraph *H* that connects all of the input nodes as a set of shortest paths, and we extracted this subgraph to visualize the results and compare NetHiKe with other methods.

### Evaluating statistical significance

To estimate the statistical significance of the nlBC values of each node, we used a Monte Carlo simulation. The same number of nodes as that on the input list was randomly sampled from the network, and the nlBC values of these nodes were calculated. After we obtained the node weight values, the weights were randomly mapped to the selected nodes. Repeating this procedure yielded an empirical distribution of the nlBC values, and we were able to calculate the simulated p-value using this distribution. Let *n* be the number of times the simulation is repeated and let *r* be the number of replicates obtained that have the centrality values ≥*nlBC*_*original*_(*v*). The simulated p-value of node *v* (*P*_*v*_) is given as follows [40]:

$$P_{v}=\frac{r+1}{n+1}.$$

In this study, we set *n *= 20,000, and the simulation count can be controlled by one of the program options.

### ErbB signaling pathway

The ErbB signaling pathway plays an important role in cell growth and cancer development [19,41]. Although the complete function of the pathway remains unknown, the ErbB signaling pathway is usually represented by the four transmembrane tyrosine kinase receptors (ERBB1 to ERBB4), several ligands of the receptors, various types of transcription factors and the complex signaling network between the receptors and the transcription factors (for example, see [42] or other pathway databases available on the web). We selected 10 ligands and 30 transcription factors from the ErbB pathway (see Additional file 1), and these molecules represent the entrance and the exit of the information flows through the pathway. In the first step of the validation, the weights of the genes were set to 1.0, and in the later step, the weight of NRG2 was calibrated from 2.0 to 20.0 for the methodology verification.

### Visualization

Although visualizing a network that includes a large number of nodes is often difficult, it is important for understanding the relationships among the nodes of interest. In this study, we visualized only the key molecules and the input genes with the subgraph containing the nodes connecting them (e.g., Figure 2). We used Cytoscape2.8.2 [43] for visualizing the network, and the Spring Embedded layout option was applied to the network to provide an overview of the relationships between the input nodes and the key molecules. For this visualization, the NetHiKe software produces input files for Cytoscape were as follows: background network information (.sif) and node attributes (.noa).

### The pathway interaction database

The Pathway Interaction Database [1,2] is a curated collection of information about known biomolecular interactions and key cellular processes assembled into signaling pathways. The database also has a web-based pathway search interface. Once the gene list is uploaded to the database, it calculates the p-values for each pathway, depending on the number of input genes that are included in the pathway. The functions of the input genes can be estimated through the output pathways with p-values; thus, we used it as a typical over-representation analysis (ORA) to grasp the approximate meanings of the input list of genes.

### Hubba

Hubba [12] is one of the most widely used network analysis programs in the molecular biology area, and we can use it through the web interface or Cytoscape plug-in. Hubba takes a network as the input data and can evaluate the importance of nodes via various methods. In this study, we used the following six methods: degree, BottleNeck, Edge Percolation Component (EPC), Maximum Neighborhood Component (MNC), Density of Maximum Neighborhood Component (DMNC), and betweenness centrality. To import our data into Hubba, we extracted the sub-network that consists of all pairs of shortest paths connecting all of the input nodes.

### GBM data from TCGA

With the recent advances in next-generation DNA sequencing technology, comprehensive cancer genome analyses are now underway [20,44]. The Cancer Genome Atlas (TCGA) is a large-scale collaborative effort to systematically characterize the genomic changes that occur in cancer by applying genome analysis technologies. TCGA is designed to target many types of cancer and to characterize various genomic changes in cancer, including somatic mutation, mRNA and miRNA expression, methylation aberration and so on. Among these data sets, glioblastoma multiforme (GBM), which is one of the most aggressive types of primary brain tumor, has been analyzed since the early stages of TCGA history. The list of genes used for this analysis was downloaded from TCGA data browser website on the TCGA data portal [45]. The TCGA data browser website has a user-friendly interface for downloading lists of genes matching many types of search conditions from the accumulated TCGA experimental results.

#### Somatic mutation data

Using the Data Portal web of TCGA, we obtained the somatic mutated genes for the following conditions: for “Disease Type”, we selected “GBM Glioblastoma multiforme”; for “Validated Somatic Mutations”, we selected “any non-silent-validated” and for Frequency ≥ 1.0%, we used the default value of the setting. We filtered out the genes that were analyzed in a small number (<100) of samples and used the mutation ratio (percentage) as the weight of each gene (Additional file 1, sheet “GBM_analysis”).

#### Expression data

In the TCGA Data Portal site, we downloaded the list of differentially expressed genes in GBM with the following conditions: “AgilentG4502A_07 log2 tumor/normal ratio” was selected for “Gene Expression”; the ratio values were set between -1.2 and 1.2, and Frequency was over 40 percent. The resulting list is available as the second sheet of Additional file 7.

### Software availability

The NetHiKe software is written in C++ and Python and is available at the following website. http://tsjshg.bitbucket.org/nethike.

Because it requires considerable system memory (4 GB or more), this software should be run on a 64-bit system.

## Abbreviations

GBM, Glioblastoma multiforme; nlBC, Node-limited betweenness centrality; NetHiKe, Network-based Hidden Key Molecule Miner; ORA, Over-representation analysis; PPI, Protein-protein interaction network; TCGA, The Cancer Genome Atlas.

## Competing interests

The authors declare that they have no competing interests.

## Authors’ contributions

ST conceived the study, performed the statistical analysis, and wrote the paper. SI and HA participated in the study’s discussions. HA helped to draft the manuscript. All of the authors read and approved the final manuscript.

## Supplementary Material

Additional file 1 The input gene list of the ErbB pathway and GBM analysis. Two input gene lists were used for this study. One contains the ligands and transcription factors from the ErbB pathway, and the other contains the mutated genes in GBM with the frequencies as the weights. (http://www.microsoft.com/download/en/details.aspx?id=10). Click here for file

Additional file 2 The collection of results for the Pathway Interaction Database analysis. The index.html file contains the links to the Pathway Interaction Database results for the various input genes. The input genes consist of the results of NetHiKe and Hubba (the top 30 genes of each). (Mini-websites, browse the index.html. Click here for file

Additional file 3 Degree, P-value, and nlBC for the ErbB pathway analysis data. Plots of the degrees, p-values, and nlBC values of genes with *P*<0*.*05 in the results of the ErbB pathway analysis (A-C) and boxplots of the nlBC values (D and E). A) Plot of the node degrees in the background network vs. nlBC. B) Degree vs. simulated p-values. C) nlBC vs. p-values. D) Boxplot visualization of the genes in Table 1. The boxes are the nlBC values generated from randomly selected genes to calculate the simulated p-values, and the yellow dots denotes the actual nlBC value that was calculated based on the input genes (listed in the Additional file 1). The simulated p-values, listed in the Table 1, are plotted as the red line associated with the right axis. E) The nlBC values that were generated by a leave-one-out method using the input genes, and the actual nlBC values as the yellow dots. The plot D and E have the same Y-axis scale (left) and the gene order in X-axis. Click here for file

Additional file 4 Comparison lists of the top 30 genes on NetHiKe and Hubba. The lists of the top 30 genes generated by NetHiKe and Hubba with the same input data. In the Hubba analysis, six different methods were used. The six-digit number indicates the Pathway Commons ID, as the molecules do not have general gene names. Click here for file

Additional file 5 The NetHiKe results of the weighted NRG2. The sheet named “NRG2_weighted_2.0” is the NetHiKe result of the input with the NRG2 weight set to 2.0, and “NRG2_weighted_20.0” is the result with the NRG2 weight set to 20.0. The genes with p-values less than 0.05 are listed. (http://www.microsoft.com/download/en/details.aspx?id=10). Click here for file

Additional file 6 The GBM network and key molecules inferred by NetHiKe. The extracted network made by genes mutated in GBM. The blue-bordered nodes are the input nodes, and the p-values are shown by the depth of the red color. Click here for file

Additional file 7 The comparison of the GBM analysis results between NetHiKe and Hubba. The sheet named “NetHiKe and Hubba results” contains the top 16 genes that were *p*<0*.*01 in the NetHiKe analysis and the same number of top-ranked genes from the various Hubba methods. There are no overlapping genes between the NetHiKe results and the Hubba results. However, there are several overlapping genes among the various Hubba methods. The second sheet, named “GBM_expression_1.2_ov40p,” contains the downloaded data from the TCA website to clarify the differentially expressed genes in the GBM analysis. (http://www.microsoft.com/download/en/details.aspx?id=10). Click here for file

Additional file 8 Two different degree distributions depend on the edge selection. Log-log degree distribution for the network constructed from the whole Pathway Commons data (A) and the selected edges (B). Click here for file
