# Supplementary material for: A simple knowledge-based mining method for exploring hidden key molecules in a human biomolecular network
Source: BMC Syst Biol. 2012 Sep 15;6:124. doi: 10.1186/1752-0509-6-124 (PMC3740779; doi:10.1186/1752-0509-6-124)
Supplement: Additional file 2 — The collection of results for the Pathway Interaction Database analysis. The index.html file contains the links to the Pathway Interaction Database results for the various input genes. The input genes consist of the results of NetHiKe and Hubba (the top 30 genes of each). (Mini-websites, browse the index.html. [file 1752-0509-6-124-S2.zip › mini_web/ErbB_NetHiKe_NRG2_x2.html]

Batch query results : Pathway Interaction Database

- Jump to main content
- Jump to navigation

---

---

- Breadcrumb trail
  1. Home
  2. Batch query
  3. Batch query results

# Batch query results for NCI-Nature Curated data (NetHiKe NRG2 weight 2.0)

| Pathway Name | Biomolecules in Group 1 | Biomolecules in Group 2 | P-value Help The pathways are ranked by the probability that they include biomolecules from the query list. The lower the p-value the greater the probability that the query list is biased towards a given pathway. The parameters for generating the p-value are the size of the query set, the number of biomolecules in a given pathway and the number of molecules in the database as a whole. |
| --- | --- | --- | --- |
| E2F transcription factor network | CDC25A, CREBBP, E2F1, E2F4, EP300, MYBL2, RB1, RBL2, RYBP, SP1, TFDP1, TFDP2 |  | 1.18e-14 |
| Regulation of retinoblastoma protein | CEBPB, CREBBP, E2F1, E2F4, EP300, ID2, JUN, RB1, TFDP1 |  | 1.84e-10 |
| Regulation of nuclear SMAD2/3 signaling | CEBPB, CREBBP, E2F4, EP300, FOXO4, JUN, SP1, TCF3, TFDP1 |  | 1.17e-09 |
| Signaling events mediated by TCPTP | CREBBP, EGFR, STAT1, STAT5A, STAT5B, STAT6 |  | 2.20e-07 |
| FoxO family signaling | CREBBP, EP300, FOXO4, RBL2, USP7, YWHAQ |  | 5.54e-07 |
| Direct p53 effectors | CREBBP, E2F1, EGFR, EP300, JUN, RB1, SP1, TFDP1 |  | 1.81e-06 |
| ErbB receptor signaling network | EGFR, ERBB2, ERBB3, ERBB4 |  | 2.43e-06 |
| FOXA1 transcription factor network | CEBPB, CREBBP, EP300, JUN, SP1 |  | 7.07e-06 |
| Regulation of nuclear beta catenin signaling and target gene transcription | EP300, ID2, JUN, TCF3, TCF7L1, YWHAQ |  | 9.58e-06 |
| Glucocorticoid receptor regulatory network | CREBBP, EP300, JUN, STAT1, STAT5A, STAT5B |  | 1.10e-05 |
| Notch-mediated HES/HEY network | CREBBP, E2F1, EP300, RB1, TCF3 |  | 1.21e-05 |
| Role of Calcineurin-dependent NFAT signaling in lymphocytes | CABIN1, CREBBP, EP300, MEF2D, YWHAQ |  | 2.76e-05 |
| IL4-mediated signaling events | CEBPB, SP1, STAT5A, STAT5B, STAT6 |  | 4.79e-05 |
| HIF-1-alpha transcription factor network | CREBBP, EP300, ID2, JUN, SP1 |  | 5.54e-05 |
| ErbB4 signaling events | ERBB2, ERBB4, STAT5A, STAT5B |  | 8.69e-05 |
| Signaling events mediated by HDAC Class III | CREBBP, EP300, FOXO4, MEF2D |  | 9.62e-05 |
| FOXM1 transcription factor network | CREBBP, EP300, RB1, SP1 |  | 1.29e-04 |
| PDGFR-beta signaling pathway | JUN, SRF, STAT1, STAT5A, STAT5B, YWHAQ |  | 1.35e-04 |
| IFN-gamma pathway | CEBPB, CREBBP, EP300, STAT1 |  | 1.41e-04 |
| a6b1 and a6b4 Integrin signaling | EGFR, ERBB2, ERBB3, YWHAQ |  | 1.83e-04 |
| p38 signaling mediated by MAPKAP kinases | SRF, TCF3, YWHAQ |  | 2.92e-04 |
| IL2-mediated signaling events | JUN, STAT1, STAT5A, STAT5B |  | 3.64e-04 |
| ErbB1 downstream signaling | EGFR, JUN, SRF, STAT1, YWHAQ |  | 5.53e-04 |
| IL3-mediated signaling events | CEBPB, STAT5A, STAT5B |  | 5.54e-04 |
| Validated nuclear estrogen receptor alpha network | CEBPB, EP300, JUN, STAT5A |  | 8.07e-04 |
| IL2 signaling events mediated by STAT5 | SP1, STAT5A, STAT5B |  | 8.45e-04 |
| Regulation of Telomerase | E2F1, EGFR, JUN, SP1 |  | 8.52e-04 |
| AP-1 transcription factor network | BAG1, EP300, JUN, SP1 |  | 9.47e-04 |
| Validated targets of C-MYC transcriptional repression | EP300, ERBB2, ID2, SP1 |  | 1.05e-03 |
| EPO signaling pathway | STAT1, STAT5A, STAT5B |  | 1.32e-03 |
| HIF-2-alpha transcription factor network | CREBBP, EP300, SP1 |  | 1.43e-03 |
| GMCSF-mediated signaling events | STAT1, STAT5A, STAT5B |  | 1.43e-03 |
| Validated transcriptional targets of AP1 family members Fra1 and Fra2 | EP300, JUN, SP1 |  | 1.55e-03 |
| C-MYB transcription factor network | CEBPB, CREBBP, EP300, SP1 |  | 1.90e-03 |
| Validated targets of C-MYC transcriptional activation | CDC25A, CREBBP, EP300, ID2 |  | 2.06e-03 |
| ErbB2/ErbB3 signaling events | ERBB2, ERBB3, JUN |  | 2.54e-03 |
| IL6-mediated signaling events | CEBPB, JUN, STAT1 |  | 3.05e-03 |
| IL5-mediated signaling events | STAT5A, STAT5B |  | 3.41e-03 |
| Signaling events mediated by PTP1B | EGFR, STAT5A, STAT5B |  | 4.04e-03 |
| Regulation of Androgen receptor activity | CREBBP, EP300, JUN |  | 4.26e-03 |
| Signaling events mediated by Stem cell factor receptor (c-Kit) | CREBBP, STAT1, STAT5A |  | 4.48e-03 |
| Validated transcriptional targets of TAp63 isoforms | EP300, SP1, YWHAQ |  | 4.71e-03 |
| FGF signaling pathway | JUN, STAT1, STAT5B |  | 5.19e-03 |
| ATF-2 transcription factor network | EP300, JUN, RB1 |  | 5.70e-03 |
| p53 pathway | CREBBP, EP300, USP7 |  | 5.97e-03 |
| IL12-mediated signaling events | STAT1, STAT5A, STAT6 |  | 8.01e-03 |
| PDGFR-alpha signaling pathway | JUN, SRF |  | 8.25e-03 |
| IL27-mediated signaling events | STAT1, STAT5A |  | 1.13e-02 |
| p73 transcription factor network | EP300, RB1, SP1 |  | 1.23e-02 |
| Calcium signaling in the CD4+ TCR pathway | CABIN1, JUN |  | 1.57e-02 |
| CD40/CD40L signaling | JUN, STAT5A |  | 1.57e-02 |
| Retinoic acid receptors-mediated signaling | CREBBP, EP300 |  | 1.57e-02 |
| EGF receptor (ErbB1) signaling pathway | EGFR, STAT1 |  | 1.96e-02 |
| IL12 signaling mediated by STAT4 | CREBBP, JUN |  | 1.96e-02 |
| Class I PI3K signaling events mediated by Akt | FOXO4, YWHAQ |  | 2.07e-02 |
| Trk receptor signaling mediated by PI3K and PLC-gamma | STAT5A, YWHAQ |  | 2.17e-02 |
| IL23-mediated signaling events | STAT1, STAT5A |  | 2.17e-02 |
| Signaling mediated by p38-alpha and p38-beta | CEBPB, JUN |  | 2.28e-02 |
| CXCR4-mediated signaling events | STAT1, STAT5A, STAT5B |  | 2.39e-02 |
| Presenilin action in Notch and Wnt signaling | CREBBP, JUN |  | 3.19e-02 |
| RhoA signaling pathway | JUN, SRF |  | 3.19e-02 |
| FOXA2 and FOXA3 transcription factor networks | CEBPB, SP1 |  | 3.31e-02 |
| Posttranslational regulation of adherens junction stability and dissassembly | CREBBP, EGFR |  | 3.44e-02 |
| Validated transcriptional targets of deltaNp63 isoforms | TCF3, TCF7L1 |  | 3.44e-02 |
| Calcineurin-regulated NFAT-dependent transcription in lymphocytes | E2F1, JUN |  | 3.56e-02 |
| Angiopoietin receptor Tie2-mediated signaling | STAT5A, STAT5B |  | 3.56e-02 |
| RAC1 signaling pathway | JUN, STAT5A |  | 4.33e-02 |
| SHP2 signaling | EGFR, STAT1 |  | 4.87e-02 |
| EGFR-dependent Endothelin signaling events | EGFR |  | 5.52e-02 |
| LPA receptor mediated events | EGFR, JUN |  | 5.71e-02 |
| Signaling events mediated by HDAC Class I | CREBBP, EP300 |  | 6.57e-02 |
| JNK signaling in the CD4+ TCR pathway | JUN |  | 8.32e-02 |
| Syndecan-3-mediated signaling events | EGFR |  | 9.92e-02 |
| E-cadherin signaling in keratinocytes | EGFR |  | 1.19e-01 |
| S1P2 pathway | JUN |  | 1.43e-01 |
| Insulin-mediated glucose transport | YWHAQ |  | 1.57e-01 |
| Osteopontin-mediated events | JUN |  | 1.66e-01 |
| Nephrin/Neph1 signaling in the kidney podocyte | JUN |  | 1.66e-01 |
| ATM pathway | CDC25A |  | 1.78e-01 |
| Trk receptor signaling mediated by the MAPK pathway | SRF |  | 1.82e-01 |
| Arf6 signaling events | EGFR |  | 1.86e-01 |
| IL1-mediated signaling events | JUN |  | 1.86e-01 |
| Signaling events mediated by HDAC Class II | SRF |  | 1.94e-01 |
| IL2 signaling events mediated by PI3K | E2F1 |  | 1.94e-01 |
| Signaling events regulated by Ret tyrosine kinase | JUN |  | 1.98e-01 |
| ATR signaling pathway | CDC25A |  | 2.02e-01 |
| Internalization of ErbB1 | EGFR |  | 2.06e-01 |
| Urokinase-type plasminogen activator (uPA) and uPAR-mediated signaling | EGFR |  | 2.09e-01 |
| Stabilization and expansion of the E-cadherin adherens junction | EGFR |  | 2.17e-01 |
| Integrin-linked kinase signaling | JUN |  | 2.24e-01 |
| TNF receptor signaling pathway | STAT1 |  | 2.30e-01 |
| Hedgehog signaling events mediated by Gli proteins | CREBBP |  | 2.30e-01 |
| LKB1 signaling events | YWHAQ |  | 2.30e-01 |
| Ceramide signaling pathway | RB1 |  | 2.37e-01 |
| Thromboxane A2 receptor signaling | EGFR |  | 2.55e-01 |
| Notch signaling pathway | EP300 |  | 2.61e-01 |
| Fc-epsilon receptor I signaling in mast cells | JUN |  | 2.70e-01 |
| Signaling events mediated by focal adhesion kinase | JUN |  | 2.75e-01 |
| Coregulation of Androgen receptor activity | SRF |  | 2.75e-01 |
| Endothelins | JUN |  | 2.77e-01 |
| BCR signaling pathway | JUN |  | 2.87e-01 |
| mTOR signaling pathway | YWHAQ |  | 2.92e-01 |
| CDC42 signaling events | JUN |  | 2.92e-01 |
| p75(NTR)-mediated signaling | E2F1 |  | 2.92e-01 |
| Downstream signaling in na�ve CD8+ T cells | JUN |  | 2.94e-01 |
| Signaling events mediated by Hepatocyte Growth Factor Receptor (c-Met) | JUN |  | 3.13e-01 |
