# Supplementary material for: A simple knowledge-based mining method for exploring hidden key molecules in a human biomolecular network
Source: BMC Syst Biol. 2012 Sep 15;6:124. doi: 10.1186/1752-0509-6-124 (PMC3740779; doi:10.1186/1752-0509-6-124)
Supplement: Additional file 2 — The collection of results for the Pathway Interaction Database analysis. The index.html file contains the links to the Pathway Interaction Database results for the various input genes. The input genes consist of the results of NetHiKe and Hubba (the top 30 genes of each). (Mini-websites, browse the index.html. [file 1752-0509-6-124-S2.zip › mini_web/ErbB_NetHiKe_NRG2_x20.html]

Batch query results : Pathway Interaction Database

- Jump to main content
- Jump to navigation

---

---

- Breadcrumb trail
  1. Home
  2. Batch query
  3. Batch query results

# Batch query results for NCI-Nature Curated data (NetHiKe NRG2 weights 20.0)

| Pathway Name | Biomolecules in Group 1 | Biomolecules in Group 2 | P-value Help The pathways are ranked by the probability that they include biomolecules from the query list. The lower the p-value the greater the probability that the query list is biased towards a given pathway. The parameters for generating the p-value are the size of the query set, the number of biomolecules in a given pathway and the number of molecules in the database as a whole. |
| --- | --- | --- | --- |
| E2F transcription factor network | BRCA1, CDC25A, CREBBP, E2F1, E2F4, EP300, MYBL2, RB1, RYBP, SP1, TFDP1, TFDP2 |  | 1.70e-12 |
| ErbB4 signaling events | ERBB2, ERBB4, ITCH, JAK2, PIK3R2, PIK3R3, STAT5A, STAT5B, YAP1 |  | 2.90e-11 |
| Signaling events mediated by TCPTP | CREBBP, EGFR, PIK3R2, PIK3R3, STAT1, STAT5A, STAT5B, STAT6 |  | 3.20e-09 |
| Regulation of retinoblastoma protein | CEBPB, CREBBP, E2F1, E2F4, EP300, ID2, JUN, RB1, TFDP1 |  | 6.12e-09 |
| PDGFR-beta signaling pathway | ARAP1, CSK, JAK2, JUN, PIK3R2, PIK3R3, SRF, STAT1, STAT5A, STAT5B, YWHAQ |  | 1.49e-08 |
| Regulation of nuclear SMAD2/3 signaling | CEBPB, CREBBP, E2F4, EP300, FOXO4, JUN, SP1, TCF3, TFDP1 |  | 3.74e-08 |
| ErbB2/ErbB3 signaling events | ERBB2, ERBB3, JAK2, JUN, PIK3R2, PIK3R3 |  | 2.35e-06 |
| FOXA1 transcription factor network | BRCA1, CEBPB, CREBBP, EP300, JUN, SP1 |  | 2.35e-06 |
| CXCR4-mediated signaling events | CSK, ITCH, JAK2, PIK3R2, PIK3R3, STAT1, STAT5A, STAT5B |  | 3.28e-06 |
| Notch-mediated HES/HEY network | CREBBP, E2F1, EP300, JAK2, RB1, TCF3 |  | 4.48e-06 |
| ErbB receptor signaling network | EGFR, ERBB2, ERBB3, ERBB4 |  | 1.06e-05 |
| IL4-mediated signaling events | CEBPB, JAK2, SP1, STAT5A, STAT5B, STAT6 |  | 2.32e-05 |
| Direct p53 effectors | CREBBP, E2F1, EGFR, EP300, JUN, RB1, SP1, TFDP1 |  | 3.08e-05 |
| p38 signaling mediated by MAPKAP kinases | MAPKAPK2, SRF, TCF3, YWHAQ |  | 3.35e-05 |
| IFN-gamma pathway | CEBPB, CREBBP, EP300, JAK2, STAT1 |  | 3.85e-05 |
| ErbB1 downstream signaling | EGFR, JUN, PIK3R2, PIK3R3, SRF, STAT1, YWHAQ |  | 5.13e-05 |
| IL6-mediated signaling events | A2M, CEBPB, JAK2, JUN, STAT1 |  | 5.93e-05 |
| Regulation of nuclear beta catenin signaling and target gene transcription | EP300, ID2, JUN, TCF3, TCF7L1, YWHAQ |  | 7.99e-05 |
| FoxO family signaling | CREBBP, EP300, FOXO4, USP7, YWHAQ |  | 7.99e-05 |
| IL3-mediated signaling events | CEBPB, JAK2, STAT5A, STAT5B |  | 8.03e-05 |
| Glucocorticoid receptor regulatory network | CREBBP, EP300, JUN, STAT1, STAT5A, STAT5B |  | 9.14e-05 |
| Signaling events mediated by PTP1B | CSK, EGFR, JAK2, STAT5A, STAT5B |  | 9.64e-05 |
| IL2-mediated signaling events | JUN, MAPKAPK2, STAT1, STAT5A, STAT5B |  | 1.26e-04 |
| Role of Calcineurin-dependent NFAT signaling in lymphocytes | CABIN1, CREBBP, EP300, MEF2D, YWHAQ |  | 1.62e-04 |
| IL5-mediated signaling events | JAK2, STAT5A, STAT5B |  | 2.47e-04 |
| EPO signaling pathway | JAK2, STAT1, STAT5A, STAT5B |  | 2.60e-04 |
| EGF receptor (ErbB1) signaling pathway | EGFR, PIK3R2, PIK3R3, STAT1 |  | 2.60e-04 |
| GMCSF-mediated signaling events | JAK2, STAT1, STAT5A, STAT5B |  | 2.90e-04 |
| HIF-1-alpha transcription factor network | CREBBP, EP300, ID2, JUN, SP1 |  | 3.16e-04 |
| Validated nuclear estrogen receptor alpha network | BRCA1, CEBPB, EP300, JUN, STAT5A |  | 3.38e-04 |
| Signaling events mediated by HDAC Class III | CREBBP, EP300, FOXO4, MEF2D |  | 3.95e-04 |
| Validated targets of C-MYC transcriptional repression | BRCA1, EP300, ERBB2, ID2, SP1 |  | 4.67e-04 |
| FOXM1 transcription factor network | CREBBP, EP300, RB1, SP1 |  | 5.24e-04 |
| p73 transcription factor network | EP300, ITCH, RB1, SP1, YAP1 |  | 6.66e-04 |
| a6b1 and a6b4 Integrin signaling | EGFR, ERBB2, ERBB3, YWHAQ |  | 7.38e-04 |
| Regulation of RhoA activity | ARAP1, ARAP3, ARHGAP4, BCR |  | 7.38e-04 |
| Validated transcriptional targets of deltaNp63 isoforms | ITCH, TCF3, TCF7L1, YAP1 |  | 8.65e-04 |
| Signaling events mediated by Stem cell factor receptor (c-Kit) | CREBBP, JAK2, STAT1, STAT5A |  | 1.34e-03 |
| Validated transcriptional targets of TAp63 isoforms | EP300, ITCH, SP1, YWHAQ |  | 1.43e-03 |
| IL27-mediated signaling events | JAK2, STAT1, STAT5A |  | 1.59e-03 |
| ATF-2 transcription factor network | BRCA1, EP300, JUN, RB1 |  | 1.84e-03 |
| IL2 signaling events mediated by STAT5 | SP1, STAT5A, STAT5B |  | 2.40e-03 |
| Nephrin/Neph1 signaling in the kidney podocyte | JUN, PIK3R2, PIK3R3 |  | 2.63e-03 |
| IL12-mediated signaling events | JAK2, STAT1, STAT5A, STAT6 |  | 2.90e-03 |
| Regulation of Telomerase | E2F1, EGFR, JUN, SP1 |  | 3.21e-03 |
| AP-1 transcription factor network | BAG1, EP300, JUN, SP1 |  | 3.55e-03 |
| HIF-2-alpha transcription factor network | CREBBP, EP300, SP1 |  | 4.00e-03 |
| IL23-mediated signaling events | JAK2, STAT1, STAT5A |  | 4.31e-03 |
| Validated transcriptional targets of AP1 family members Fra1 and Fra2 | EP300, JUN, SP1 |  | 4.31e-03 |
| Signaling mediated by p38-alpha and p38-beta | CEBPB, JUN, MAPKAPK2 |  | 4.64e-03 |
| Internalization of ErbB1 | EGFR, PIK3R2, PIK3R3 |  | 5.71e-03 |
| C-MYB transcription factor network | CEBPB, CREBBP, EP300, SP1 |  | 6.81e-03 |
| Validated targets of C-MYC transcriptional activation | CDC25A, CREBBP, EP300, ID2 |  | 7.35e-03 |
| Calcineurin-regulated NFAT-dependent transcription in lymphocytes | E2F1, ITCH, JUN |  | 9.20e-03 |
| Class I PI3K signaling events | ARAP3, PIK3R2, PIK3R3 |  | 9.20e-03 |
| Regulation of Androgen receptor activity | CREBBP, EP300, JUN |  | 1.13e-02 |
| FGF signaling pathway | JUN, STAT1, STAT5B |  | 1.36e-02 |
| SHP2 signaling | EGFR, JAK2, STAT1 |  | 1.49e-02 |
| p53 pathway | CREBBP, EP300, USP7 |  | 1.55e-02 |
| PDGFR-alpha signaling pathway | JUN, SRF |  | 1.63e-02 |
| BCR signaling pathway | BCR, CSK, JUN |  | 2.12e-02 |
| TRAIL signaling pathway | PIK3R2, PIK3R3 |  | 2.52e-02 |
| Insulin-mediated glucose transport | TRIP10, YWHAQ |  | 2.68e-02 |
| Calcium signaling in the CD4+ TCR pathway | CABIN1, JUN |  | 3.02e-02 |
| CD40/CD40L signaling | JUN, STAT5A |  | 3.02e-02 |
| Retinoic acid receptors-mediated signaling | CREBBP, EP300 |  | 3.02e-02 |
| ATM pathway | BRCA1, CDC25A |  | 3.55e-02 |
| IL12 signaling mediated by STAT4 | CREBBP, JUN |  | 3.73e-02 |
| Trk receptor signaling mediated by the MAPK pathway | MAPKAPK2, SRF |  | 3.73e-02 |
| Class I PI3K signaling events mediated by Akt | FOXO4, YWHAQ |  | 3.91e-02 |
| Trk receptor signaling mediated by PI3K and PLC-gamma | STAT5A, YWHAQ |  | 4.10e-02 |
| Regulation of RAC1 activity | BCR, RALBP1 |  | 4.29e-02 |
| FAS (CD95) signaling pathway | PIK3R2, PIK3R3 |  | 4.48e-02 |
| CXCR3-mediated signaling events | PIK3R2, PIK3R3 |  | 5.47e-02 |
| Presenilin action in Notch and Wnt signaling | CREBBP, JUN |  | 5.87e-02 |
| RhoA signaling pathway | JUN, SRF |  | 5.87e-02 |
| FOXA2 and FOXA3 transcription factor networks | CEBPB, SP1 |  | 6.08e-02 |
| Posttranslational regulation of adherens junction stability and dissassembly | CREBBP, EGFR |  | 6.29e-02 |
| Angiopoietin receptor Tie2-mediated signaling | STAT5A, STAT5B |  | 6.50e-02 |
| EGFR-dependent Endothelin signaling events | EGFR |  | 7.76e-02 |
| TGF-beta receptor signaling | ITCH, YAP1 |  | 7.77e-02 |
| RAC1 signaling pathway | JUN, STAT5A |  | 7.77e-02 |
| Notch signaling pathway | EP300, ITCH |  | 8.42e-02 |
| Coregulation of Androgen receptor activity | BRCA1, SRF |  | 9.51e-02 |
| Endothelins | JAK2, JUN |  | 9.73e-02 |
| LPA receptor mediated events | EGFR, JUN |  | 9.95e-02 |
| Signaling events mediated by HDAC Class I | CREBBP, EP300 |  | 1.13e-01 |
| JNK signaling in the CD4+ TCR pathway | JUN |  | 1.15e-01 |
| Syndecan-3-mediated signaling events | EGFR |  | 1.36e-01 |
| E-cadherin signaling in keratinocytes | EGFR |  | 1.62e-01 |
| S1P2 pathway | JUN |  | 1.92e-01 |
| S1P3 pathway | JAK2 |  | 2.08e-01 |
| BARD1 signaling events | BRCA1 |  | 2.13e-01 |
| Osteopontin-mediated events | JUN |  | 2.19e-01 |
| Regulation of CDC42 activity | RALBP1 |  | 2.19e-01 |
| Aurora A signaling | BRCA1 |  | 2.24e-01 |
| Arf6 signaling events | EGFR |  | 2.42e-01 |
| IL1-mediated signaling events | JUN |  | 2.42e-01 |
| Signaling events mediated by HDAC Class II | SRF |  | 2.51e-01 |
| IL2 signaling events mediated by PI3K | E2F1 |  | 2.51e-01 |
| Signaling events regulated by Ret tyrosine kinase | JUN |  | 2.55e-01 |
| ATR signaling pathway | CDC25A |  | 2.60e-01 |
| Urokinase-type plasminogen activator (uPA) and uPAR-mediated signaling | EGFR |  | 2.68e-01 |
| Stabilization and expansion of the E-cadherin adherens junction | EGFR |  | 2.75e-01 |
| Insulin Pathway | TRIP10 |  | 2.79e-01 |
| Integrin-linked kinase signaling | JUN |  | 2.83e-01 |
| TNF receptor signaling pathway | STAT1 |  | 2.89e-01 |
| Hedgehog signaling events mediated by Gli proteins | CREBBP |  | 2.89e-01 |
| LKB1 signaling events | YWHAQ |  | 2.89e-01 |
| Fanconi anemia pathway | BRCA1 |  | 2.93e-01 |
| Ceramide signaling pathway | RB1 |  | 2.96e-01 |
| Thromboxane A2 receptor signaling | EGFR |  | 3.14e-01 |
| TCR signaling in na�ve CD8+ T cells | CSK |  | 3.14e-01 |
| Fc-epsilon receptor I signaling in mast cells | JUN |  | 3.26e-01 |
| Signaling events mediated by focal adhesion kinase | JUN |  | 3.31e-01 |
| TCR signaling in na�ve CD4+ T cells | CSK |  | 3.41e-01 |
| mTOR signaling pathway | YWHAQ |  | 3.45e-01 |
| CDC42 signaling events | JUN |  | 3.45e-01 |
| p75(NTR)-mediated signaling | E2F1 |  | 3.45e-01 |
| Downstream signaling in na�ve CD8+ T cells | JUN |  | 3.46e-01 |
| Signaling events mediated by VEGFR1 and VEGFR2 | MAPKAPK2 |  | 3.50e-01 |
| Signaling events mediated by Hepatocyte Growth Factor Receptor (c-Met) | JUN |  | 3.59e-01 |
