# Supplementary material for: A simple knowledge-based mining method for exploring hidden key molecules in a human biomolecular network
Source: BMC Syst Biol. 2012 Sep 15;6:124. doi: 10.1186/1752-0509-6-124 (PMC3740779; doi:10.1186/1752-0509-6-124)
Supplement: Additional file 2 — The collection of results for the Pathway Interaction Database analysis. The index.html file contains the links to the Pathway Interaction Database results for the various input genes. The input genes consist of the results of NetHiKe and Hubba (the top 30 genes of each). (Mini-websites, browse the index.html. [file 1752-0509-6-124-S2.zip › mini_web/Hubba_bc.html]

Batch query results : Pathway Interaction Database

- Jump to main content
- Jump to navigation

---

---

- Breadcrumb trail
  1. Home
  2. Batch query
  3. Batch query results

# Batch query results for NCI-Nature Curated data (Hubba betweenness centrality)

| Pathway Name | Biomolecules in Group 1 | Biomolecules in Group 2 | P-value Help The pathways are ranked by the probability that they include biomolecules from the query list. The lower the p-value the greater the probability that the query list is biased towards a given pathway. The parameters for generating the p-value are the size of the query set, the number of biomolecules in a given pathway and the number of molecules in the database as a whole. |
| --- | --- | --- | --- |
| Glucocorticoid receptor regulatory network | CREB1, CREBBP, EP300, HDAC1, HDAC2, HSP90AA1, JUN, MAPK1, STAT1, STAT5A, STAT5B, TP53 |  | 3.58e-14 |
| Regulation of nuclear SMAD2/3 signaling | AR, CREB1, CREBBP, E2F4, EP300, ESR1, HDAC1, HDAC2, JUN, SMAD4, SP1 |  | 1.20e-12 |
| Signaling events mediated by TCPTP | CREBBP, EGF, EGFR, SRC, STAT1, STAT3, STAT5A, STAT5B, STAT6 |  | 2.71e-12 |
| Regulation of Telomerase | E2F1, EGF, EGFR, ESR1, HDAC1, HDAC2, HSP90AA1, JUN, MAPK1, SP1 |  | 6.84e-12 |
| Signaling events mediated by PTP1B | EGF, EGFR, FYN, JAK2, LYN, SRC, STAT3, STAT5A, STAT5B |  | 1.70e-11 |
| Notch-mediated HES/HEY network | AR, CREBBP, E2F1, EP300, HDAC1, JAK2, RB1, STAT3 |  | 4.30e-10 |
| GMCSF-mediated signaling events | JAK2, LYN, MAPK1, STAT1, STAT3, STAT5A, STAT5B |  | 1.66e-09 |
| PDGFR-beta signaling pathway | FYN, JAK2, JUN, LYN, MAPK1, SRC, STAT1, STAT3, STAT5A, STAT5B |  | 4.07e-09 |
| E2F transcription factor network | CDK1, CREBBP, E2F1, E2F4, EP300, HDAC1, RB1, SP1 |  | 1.57e-08 |
| Regulation of Androgen receptor activity | AR, CREBBP, EP300, HDAC1, HSP90AA1, JUN, SRC |  | 2.81e-08 |
| IL2-mediated signaling events | FYN, JUN, MAPK1, STAT1, STAT3, STAT5A, STAT5B |  | 3.67e-08 |
| EGF receptor (ErbB1) signaling pathway | EGF, EGFR, MAPK1, SRC, STAT1, STAT3 |  | 6.12e-08 |
| ErbB4 signaling events | ERBB4, FYN, JAK2, MAPK1, STAT5A, STAT5B |  | 1.02e-07 |
| Direct p53 effectors | CREBBP, E2F1, EGFR, EP300, HDAC2, JUN, RB1, SP1, TP53 |  | 1.33e-07 |
| Regulation of retinoblastoma protein | CREBBP, E2F1, E2F4, EP300, HDAC1, JUN, RB1 |  | 1.48e-07 |
| CXCR4-mediated signaling events | FYN, JAK2, LYN, SRC, STAT1, STAT3, STAT5A, STAT5B |  | 1.74e-07 |
| FOXM1 transcription factor network | CDK1, CREBBP, EP300, ESR1, RB1, SP1 |  | 1.91e-07 |
| IFN-gamma pathway | CREBBP, EP300, JAK2, MAPK1, STAT1, STAT3 |  | 2.20e-07 |
| AP-1 transcription factor network | CDK1, CREB1, EP300, ESR1, JUN, SP1, TP53 |  | 2.21e-07 |
| FOXA1 transcription factor network | AR, CREBBP, EP300, ESR1, JUN, SP1 |  | 2.54e-07 |
| ErbB1 downstream signaling | CREB1, EGF, EGFR, JUN, MAPK1, SRC, STAT1, STAT3 |  | 2.89e-07 |
| Signaling events mediated by Stem cell factor receptor (c-Kit) | CREBBP, JAK2, LYN, STAT1, STAT3, STAT5A |  | 8.81e-07 |
| Retinoic acid receptors-mediated signaling | CDK1, CREBBP, EP300, HDAC1, MAPK1 |  | 1.19e-06 |
| IL5-mediated signaling events | JAK2, LYN, STAT5A, STAT5B |  | 1.35e-06 |
| ATF-2 transcription factor network | CREB1, EP300, ESR1, JUN, MAPK1, RB1 |  | 1.50e-06 |
| EPO signaling pathway | JAK2, LYN, STAT1, STAT5A, STAT5B |  | 2.22e-06 |
| ErbB receptor signaling network | EGF, EGFR, ERBB4, HSP90AA1 |  | 2.43e-06 |
| HIF-1-alpha transcription factor network | CREB1, CREBBP, EP300, JUN, SMAD4, SP1 |  | 3.18e-06 |
| Validated nuclear estrogen receptor alpha network | EP300, ESR1, HDAC1, JUN, SMAD4, STAT5A |  | 3.46e-06 |
| ErbB2/ErbB3 signaling events | JAK2, JUN, MAPK1, SRC, STAT3 |  | 7.07e-06 |
| Posttranslational regulation of adherens junction stability and dissassembly | CREBBP, EGF, EGFR, FYN, SRC |  | 1.09e-05 |
| LKB1 signaling events | CREB1, ESR1, HSP90AA1, SMAD4, TP53 |  | 1.09e-05 |
| IL3-mediated signaling events | HDAC1, JAK2, STAT5A, STAT5B |  | 1.88e-05 |
| IL27-mediated signaling events | JAK2, STAT1, STAT3, STAT5A |  | 1.88e-05 |
| Thromboxane A2 receptor signaling | EGF, EGFR, FYN, LYN, SRC |  | 2.33e-05 |
| FGF signaling pathway | JUN, MAPK1, SRC, STAT1, STAT5B |  | 2.54e-05 |
| Nongenotropic Androgen signaling | AR, CREB1, MAPK1, SRC |  | 3.85e-05 |
| Alpha-synuclein signaling | FYN, LYN, MAPK1, SRC |  | 4.38e-05 |
| IL4-mediated signaling events | JAK2, SP1, STAT5A, STAT5B, STAT6 |  | 4.79e-05 |
| IL12-mediated signaling events | JAK2, STAT1, STAT3, STAT5A, STAT6 |  | 5.54e-05 |
| Signaling events mediated by HDAC Class I | CREBBP, EP300, HDAC1, HDAC2, STAT3 |  | 7.30e-05 |
| IL23-mediated signaling events | JAK2, STAT1, STAT3, STAT5A |  | 7.82e-05 |
| Validated targets of C-MYC transcriptional repression | CREB1, EP300, HDAC1, SMAD4, SP1 |  | 8.33e-05 |
| Signaling mediated by p38-alpha and p38-beta | CREB1, ESR1, JUN, TP53 |  | 8.69e-05 |
| Signaling events regulated by Ret tyrosine kinase | CREB1, JUN, MAPK1, SRC |  | 9.62e-05 |
| Regulation of nuclear beta catenin signaling and target gene transcription | AR, EP300, HDAC1, HDAC2, JUN |  | 1.36e-04 |
| Syndecan-3-mediated signaling events | EGFR, FYN, SRC |  | 1.53e-04 |
| Presenilin action in Notch and Wnt signaling | CREBBP, HDAC1, JUN, MAPK1 |  | 1.83e-04 |
| IL6-mediated signaling events | JAK2, JUN, STAT1, STAT3 |  | 1.99e-04 |
| Validated targets of C-MYC transcriptional activation | CREBBP, EP300, HSP90AA1, SMAD4, TP53 |  | 2.00e-04 |
| Class I PI3K signaling events | FYN, HSP90AA1, LYN, SRC |  | 2.34e-04 |
| Angiopoietin receptor Tie2-mediated signaling | FYN, MAPK1, STAT5A, STAT5B |  | 2.34e-04 |
| Ceramide signaling pathway | EGF, MAPK1, RB1, TRAF2 |  | 2.53e-04 |
| E-cadherin signaling in keratinocytes | EGFR, FYN, SRC |  | 2.92e-04 |
| SHP2 signaling | EGF, EGFR, JAK2, STAT1 |  | 4.75e-04 |
| p53 pathway | CREBBP, EP300, TP53, USP7 |  | 5.06e-04 |
| Fc-epsilon receptor I signaling in mast cells | FYN, JUN, LYN, MAPK1 |  | 5.38e-04 |
| Signaling events mediated by focal adhesion kinase | FYN, JUN, MAPK1, SRC |  | 6.08e-04 |
| Glypican 1 network | FYN, LYN, SRC |  | 6.20e-04 |
| Endothelins | JAK2, JUN, MAPK1, SRC |  | 6.44e-04 |
| LPA receptor mediated events | EGFR, JUN, LYN, SRC |  | 6.83e-04 |
| S1P3 pathway | JAK2, MAPK1, SRC |  | 7.65e-04 |
| Ephrin B reverse signaling | FYN, LYN, SRC |  | 8.45e-04 |
| IL2 signaling events mediated by STAT5 | SP1, STAT5A, STAT5B |  | 8.45e-04 |
| CD40/CD40L signaling | JUN, STAT5A, TRAF2 |  | 9.30e-04 |
| Regulation of p38-alpha and p38-beta | FYN, LYN, SRC |  | 9.30e-04 |
| Netrin-mediated signaling events | FYN, MAPK1, SRC |  | 1.02e-03 |
| Signaling events mediated by VEGFR1 and VEGFR2 | FYN, HSP90AA1, MAPK1, SRC |  | 1.05e-03 |
| EPHA forward signaling | FYN, LYN, SRC |  | 1.22e-03 |
| IL12 signaling mediated by STAT4 | CREBBP, JUN, STAT3 |  | 1.32e-03 |
| EGFR-dependent Endothelin signaling events | EGF, EGFR |  | 1.39e-03 |
| p73 transcription factor network | CDK1, EP300, RB1, SP1 |  | 1.40e-03 |
| HIF-2-alpha transcription factor network | CREBBP, EP300, SP1 |  | 1.43e-03 |
| Arf6 signaling events | EGF, EGFR, SRC |  | 1.43e-03 |
| Trk receptor signaling mediated by PI3K and PLC-gamma | CREB1, SRC, STAT5A |  | 1.55e-03 |
| Validated transcriptional targets of AP1 family members Fra1 and Fra2 | EP300, JUN, SP1 |  | 1.55e-03 |
| Signaling events mediated by HDAC Class III | CREBBP, EP300, TP53 |  | 1.80e-03 |
| amb2 Integrin signaling | FYN, LYN, SRC |  | 2.08e-03 |
| Internalization of ErbB1 | EGF, EGFR, SRC |  | 2.08e-03 |
| Integrin-linked kinase signaling | CREB1, HSP90AA1, JUN |  | 2.88e-03 |
| Hedgehog signaling events mediated by Gli proteins | CREBBP, HDAC1, HDAC2 |  | 3.24e-03 |
| FoxO family signaling | CREBBP, EP300, USP7 |  | 3.63e-03 |
| RAC1 signaling pathway | JUN, STAT3, STAT5A |  | 4.71e-03 |
| Hypoxic and oxygen homeostasis regulation of HIF-1-alpha | HSP90AA1, TP53 |  | 6.22e-03 |
| Regulation of cytoplasmic and nuclear SMAD2/3 signaling | MAPK1, SMAD4 |  | 6.87e-03 |
| BCR signaling pathway | JUN, LYN, MAPK1 |  | 8.33e-03 |
| Signaling events mediated by PRL | MAPK1, SRC |  | 8.98e-03 |
| CDC42 signaling events | JUN, MAPK1, SRC |  | 8.99e-03 |
| VEGFR3 signaling in lymphatic endothelium | CREB1, MAPK1 |  | 1.05e-02 |
| S1P2 pathway | JUN, MAPK1 |  | 1.13e-02 |
| Integrins in angiogenesis | HSP90AA1, MAPK1, SRC |  | 1.19e-02 |
| ALK1 signaling events | MAPK1, SMAD4 |  | 1.22e-02 |
| Signaling events mediated by Hepatocyte Growth Factor Receptor (c-Met) | JUN, MAPK1, SRC |  | 1.27e-02 |
| TRAIL signaling pathway | MAPK1, TRAF2 |  | 1.30e-02 |
| VEGFR1 specific signals | HSP90AA1, MAPK1 |  | 1.48e-02 |
| C-MYB transcription factor network | CREBBP, EP300, SP1 |  | 1.53e-02 |
| Osteopontin-mediated events | JUN, MAPK1 |  | 1.57e-02 |
| Nephrin/Neph1 signaling in the kidney podocyte | FYN, JUN |  | 1.57e-02 |
| Syndecan-2-mediated signaling events | MAPK1, SRC |  | 1.76e-02 |
| Ephrin A reverse signaling | FYN |  | 1.91e-02 |
| Trk receptor signaling mediated by the MAPK pathway | CREB1, MAPK1 |  | 1.96e-02 |
| Class I PI3K signaling events mediated by Akt | HSP90AA1, SRC |  | 2.07e-02 |
| Signaling events mediated by HDAC Class II | ESR1, HSP90AA1 |  | 2.28e-02 |
| IL2 signaling events mediated by PI3K | E2F1, HSP90AA1 |  | 2.28e-02 |
| EPHB forward signaling | MAPK1, SRC |  | 2.50e-02 |
| Urokinase-type plasminogen activator (uPA) and uPAR-mediated signaling | EGFR, SRC |  | 2.72e-02 |
| Plasma membrane estrogen receptor signaling | ESR1, SRC |  | 2.72e-02 |
| BMP receptor signaling | MAPK1, SMAD4 |  | 2.84e-02 |
| CXCR3-mediated signaling events | MAPK1, SRC |  | 2.95e-02 |
| Stabilization and expansion of the E-cadherin adherens junction | EGF, EGFR |  | 2.95e-02 |
| a6b1 and a6b4 Integrin signaling | EGF, EGFR |  | 3.19e-02 |
| FOXA2 and FOXA3 transcription factor networks | CREB1, SP1 |  | 3.31e-02 |
| TNF receptor signaling pathway | STAT1, TRAF2 |  | 3.44e-02 |
| Calcineurin-regulated NFAT-dependent transcription in lymphocytes | E2F1, JUN |  | 3.56e-02 |
| PLK3 signaling events | TP53 |  | 3.75e-02 |
| Class IB PI3K non-lipid kinase events | MAPK1 |  | 3.75e-02 |
| Caspase cascade in apoptosis | TRAF2, VIM |  | 3.94e-02 |
| Validated transcriptional targets of TAp63 isoforms | EP300, SP1 |  | 4.33e-02 |
| Role of Calcineurin-dependent NFAT signaling in lymphocytes | CREBBP, EP300 |  | 4.74e-02 |
| Notch signaling pathway | EP300, HDAC1 |  | 4.74e-02 |
| Neurotrophic factor-mediated Trk receptor signaling | MAPK1, STAT3 |  | 5.29e-02 |
| p75(NTR)-mediated signaling | E2F1, TP53 |  | 6.43e-02 |
| Downstream signaling in na�ve CD8+ T cells | JUN, MAPK1 |  | 6.57e-02 |
| ALK2 signaling events | SMAD4 |  | 6.67e-02 |
| JNK signaling in the CD4+ TCR pathway | JUN |  | 8.32e-02 |
| S1P4 pathway | MAPK1 |  | 8.32e-02 |
| Ras signaling in the CD4+ TCR pathway | MAPK1 |  | 8.32e-02 |
| Sumoylation by RanBP2 regulates transcriptional repression | HDAC1 |  | 8.86e-02 |
| Arf6 downstream pathway | MAPK1 |  | 8.86e-02 |
| LPA4-mediated signaling events | CREB1 |  | 9.39e-02 |
| Atypical NF-kappaB pathway | SRC |  | 9.92e-02 |
| EPHA2 forward signaling | SRC |  | 1.09e-01 |
| S1P1 pathway | MAPK1 |  | 1.19e-01 |
| p38 signaling mediated by MAPKAP kinases | CREB1 |  | 1.19e-01 |
| PDGFR-alpha signaling pathway | JUN |  | 1.24e-01 |
| Cellular roles of Anthrax toxin | MAPK1 |  | 1.24e-01 |
| Alpha9 beta1 integrin signaling events | SRC |  | 1.39e-01 |
| IL8- and CXCR1-mediated signaling events | LYN |  | 1.57e-01 |
| Reelin signaling pathway | FYN |  | 1.57e-01 |
| Nectin adhesion pathway | SRC |  | 1.61e-01 |
| p38 MAPK signaling pathway | TRAF2 |  | 1.61e-01 |
| BARD1 signaling events | TP53 |  | 1.61e-01 |
| Calcium signaling in the CD4+ TCR pathway | JUN |  | 1.66e-01 |
| Aurora A signaling | TP53 |  | 1.70e-01 |
| Alpha4 beta1 integrin signaling events | SRC |  | 1.78e-01 |
| IL8- and CXCR2-mediated signaling events | LYN |  | 1.82e-01 |
| HIV-1 Nef: Negative effector of Fas and TNF-alpha | TRAF2 |  | 1.82e-01 |
| IL1-mediated signaling events | JUN |  | 1.86e-01 |
| Aurora B signaling | VIM |  | 1.98e-01 |
| FAS (CD95) signaling pathway | SRC |  | 1.98e-01 |
| E-cadherin signaling in the nascent adherens junction | SRC |  | 2.02e-01 |
| PLK1 signaling events | CDK1 |  | 2.24e-01 |
| RhoA signaling pathway | JUN |  | 2.24e-01 |
| Syndecan-1-mediated signaling events | MAPK1 |  | 2.34e-01 |
| TGF-beta receptor signaling | SMAD4 |  | 2.52e-01 |
| TCR signaling in na�ve CD8+ T cells | FYN |  | 2.55e-01 |
| Coregulation of Androgen receptor activity | AR |  | 2.75e-01 |
| TCR signaling in na�ve CD4+ T cells | FYN |  | 2.87e-01 |
| mTOR signaling pathway | MAPK1 |  | 2.92e-01 |
