# Supplementary material for: A simple knowledge-based mining method for exploring hidden key molecules in a human biomolecular network
Source: BMC Syst Biol. 2012 Sep 15;6:124. doi: 10.1186/1752-0509-6-124 (PMC3740779; doi:10.1186/1752-0509-6-124)
Supplement: Additional file 2 — The collection of results for the Pathway Interaction Database analysis. The index.html file contains the links to the Pathway Interaction Database results for the various input genes. The input genes consist of the results of NetHiKe and Hubba (the top 30 genes of each). (Mini-websites, browse the index.html. [file 1752-0509-6-124-S2.zip › mini_web/Hubba_bn.html]

Batch query results : Pathway Interaction Database

- Jump to main content
- Jump to navigation

---

---

- Breadcrumb trail
  1. Home
  2. Batch query
  3. Batch query results

# Batch query results for NCI-Nature Curated data (Hubba bottle neck)

| Pathway Name | Biomolecules in Group 1 | Biomolecules in Group 2 | P-value Help The pathways are ranked by the probability that they include biomolecules from the query list. The lower the p-value the greater the probability that the query list is biased towards a given pathway. The parameters for generating the p-value are the size of the query set, the number of biomolecules in a given pathway and the number of molecules in the database as a whole. |
| --- | --- | --- | --- |
| Glucocorticoid receptor regulatory network | CDKN1A, CREB1, CREBBP, EP300, HSP90AA1, JUN, NR3C1, STAT1, STAT5A, STAT5B, TBP, TP53 |  | 5.55e-14 |
| Regulation of nuclear SMAD2/3 signaling | AR, CDKN1A, CREB1, CREBBP, EP300, ESR1, JUN, NR3C1, SMAD4, SP1, TCF3 |  | 1.78e-12 |
| Signaling events mediated by TCPTP | CREBBP, EGFR, PIK3R1, SRC, STAT1, STAT3, STAT5A, STAT5B, STAT6 |  | 3.71e-12 |
| Signaling events mediated by PTP1B | EGFR, FYN, LYN, PIK3R1, SRC, STAT3, STAT5A, STAT5B |  | 9.25e-10 |
| Notch-mediated HES/HEY network | AR, CREBBP, E2F1, EP300, RB1, STAT3, TCF3 |  | 2.01e-08 |
| Regulation of Androgen receptor activity | AR, CREBBP, EP300, HSP90AA1, JUN, NR3C1, SRC |  | 3.54e-08 |
| IL2-mediated signaling events | FYN, JUN, PIK3R1, STAT1, STAT3, STAT5A, STAT5B |  | 4.61e-08 |
| GMCSF-mediated signaling events | LYN, PIK3R1, STAT1, STAT3, STAT5A, STAT5B |  | 8.88e-08 |
| PDGFR-beta signaling pathway | FYN, JUN, LYN, PIK3R1, SRC, STAT1, STAT3, STAT5A, STAT5B |  | 9.42e-08 |
| Direct p53 effectors | CDKN1A, CREBBP, E2F1, EGFR, EP300, JUN, RB1, SP1, TP53 |  | 1.78e-07 |
| Regulation of retinoblastoma protein | CDKN1A, CREBBP, E2F1, EP300, JUN, RB1, TBP |  | 1.85e-07 |
| CXCR4-mediated signaling events | FYN, LYN, PIK3R1, SRC, STAT1, STAT3, STAT5A, STAT5B |  | 2.25e-07 |
| AP-1 transcription factor network | CREB1, EP300, ESR1, JUN, NR3C1, SP1, TP53 |  | 2.77e-07 |
| FOXA1 transcription factor network | AR, CREBBP, EP300, ESR1, JUN, SP1 |  | 3.08e-07 |
| Signaling events mediated by Stem cell factor receptor (c-Kit) | CREBBP, LYN, PIK3R1, STAT1, STAT3, STAT5A |  | 1.07e-06 |
| Validated targets of C-MYC transcriptional activation | BMI1, COMMD3-BMI1, CREBBP, EP300, HSP90AA1, SMAD4, TP53 |  | 1.21e-06 |
| IL5-mediated signaling events | LYN, PIK3R1, STAT5A, STAT5B |  | 1.53e-06 |
| EPO signaling pathway | LYN, PIK3R1, STAT1, STAT5A, STAT5B |  | 2.60e-06 |
| EGF receptor (ErbB1) signaling pathway | EGFR, PIK3R1, SRC, STAT1, STAT3 |  | 2.60e-06 |
| HIF-1-alpha transcription factor network | CREB1, CREBBP, EP300, JUN, SMAD4, SP1 |  | 3.83e-06 |
| ErbB4 signaling events | ERBB4, FYN, PIK3R1, STAT5A, STAT5B |  | 3.96e-06 |
| Regulation of Telomerase | E2F1, EGFR, ESR1, HSP90AA1, JUN, SP1 |  | 4.55e-06 |
| ErbB1 downstream signaling | CREB1, EGFR, JUN, PIK3R1, SRC, STAT1, STAT3 |  | 5.39e-06 |
| Validated targets of C-MYC transcriptional repression | CDKN1A, CREB1, EP300, SMAD4, SP1, TBP |  | 6.33e-06 |
| FOXM1 transcription factor network | CREBBP, EP300, ESR1, RB1, SP1 |  | 6.55e-06 |
| IFN-gamma pathway | CREBBP, EP300, PIK3R1, STAT1, STAT3 |  | 7.37e-06 |
| E2F transcription factor network | CDKN1A, CREBBP, E2F1, EP300, RB1, SP1 |  | 7.99e-06 |
| E-cadherin signaling in keratinocytes | EGFR, FYN, PIK3R1, SRC |  | 8.80e-06 |
| LKB1 signaling events | CREB1, ESR1, HSP90AA1, SMAD4, TP53 |  | 1.27e-05 |
| Class I PI3K signaling events | FYN, HSP90AA1, LYN, PIK3R1, SRC |  | 1.41e-05 |
| Angiopoietin receptor Tie2-mediated signaling | CDKN1A, FYN, PIK3R1, STAT5A, STAT5B |  | 1.41e-05 |
| FGF signaling pathway | JUN, PIK3R1, SRC, STAT1, STAT5B |  | 2.96e-05 |
| ATF-2 transcription factor network | CREB1, EP300, ESR1, JUN, RB1 |  | 3.50e-05 |
| Ephrin B reverse signaling | FYN, LYN, PIK3R1, SRC |  | 3.82e-05 |
| IL2 signaling events mediated by STAT5 | PIK3R1, SP1, STAT5A, STAT5B |  | 3.82e-05 |
| Nongenotropic Androgen signaling | AR, CREB1, PIK3R1, SRC |  | 4.36e-05 |
| LPA receptor mediated events | EGFR, JUN, LYN, PIK3R1, SRC |  | 5.58e-05 |
| IL4-mediated signaling events | PIK3R1, SP1, STAT5A, STAT5B, STAT6 |  | 5.58e-05 |
| Validated nuclear estrogen receptor alpha network | EP300, ESR1, JUN, SMAD4, STAT5A |  | 6.92e-05 |
| Trk receptor signaling mediated by PI3K and PLC-gamma | CREB1, PIK3R1, SRC, STAT5A |  | 8.83e-05 |
| IL23-mediated signaling events | PIK3R1, STAT1, STAT3, STAT5A |  | 8.83e-05 |
| Signaling mediated by p38-alpha and p38-beta | CREB1, ESR1, JUN, TP53 |  | 9.82e-05 |
| Signaling events mediated by HDAC Class III | CDKN1A, CREBBP, EP300, TP53 |  | 1.09e-04 |
| Signaling events regulated by Ret tyrosine kinase | CREB1, JUN, PIK3R1, SRC |  | 1.09e-04 |
| ErbB receptor signaling network | EGFR, ERBB4, HSP90AA1 |  | 1.39e-04 |
| Regulation of nuclear beta catenin signaling and target gene transcription | AR, EP300, JUN, TCF3, TCF7L1 |  | 1.58e-04 |
| Syndecan-3-mediated signaling events | EGFR, FYN, SRC |  | 1.68e-04 |
| ErbB2/ErbB3 signaling events | JUN, PIK3R1, SRC, STAT3 |  | 1.74e-04 |
| IL6-mediated signaling events | JUN, PIK3R1, STAT1, STAT3 |  | 2.25e-04 |
| Posttranslational regulation of adherens junction stability and dissassembly | CREBBP, EGFR, FYN, SRC |  | 2.44e-04 |
| Thromboxane A2 receptor signaling | EGFR, FYN, LYN, SRC |  | 4.39e-04 |
| p53 pathway | CREBBP, EP300, TP53, USP7 |  | 5.69e-04 |
| Fc-epsilon receptor I signaling in mast cells | FYN, JUN, LYN, PIK3R1 |  | 6.06e-04 |
| IL3-mediated signaling events | PIK3R1, STAT5A, STAT5B |  | 6.07e-04 |
| IL27-mediated signaling events | STAT1, STAT3, STAT5A |  | 6.07e-04 |
| Glypican 1 network | FYN, LYN, SRC |  | 6.78e-04 |
| Signaling events mediated by focal adhesion kinase | FYN, JUN, PIK3R1, SRC |  | 6.83e-04 |
| IL12-mediated signaling events | STAT1, STAT3, STAT5A, STAT6 |  | 8.58e-04 |
| CD40/CD40L signaling | JUN, STAT5A, TRAF2 |  | 1.02e-03 |
| Regulation of p38-alpha and p38-beta | FYN, LYN, SRC |  | 1.02e-03 |
| Nephrin/Neph1 signaling in the kidney podocyte | FYN, JUN, PIK3R1 |  | 1.02e-03 |
| Alpha-synuclein signaling | FYN, LYN, SRC |  | 1.12e-03 |
| Netrin-mediated signaling events | FYN, PIK3R1, SRC |  | 1.12e-03 |
| Signaling events mediated by VEGFR1 and VEGFR2 | FYN, HSP90AA1, PIK3R1, SRC |  | 1.18e-03 |
| EPHA forward signaling | FYN, LYN, SRC |  | 1.33e-03 |
| IL12 signaling mediated by STAT4 | CREBBP, JUN, STAT3 |  | 1.45e-03 |
| Class I PI3K signaling events mediated by Akt | CDKN1A, HSP90AA1, SRC |  | 1.57e-03 |
| HIF-2-alpha transcription factor network | CREBBP, EP300, SP1 |  | 1.57e-03 |
| p73 transcription factor network | CDKN1A, EP300, RB1, SP1 |  | 1.57e-03 |
| Validated transcriptional targets of AP1 family members Fra1 and Fra2 | EP300, JUN, SP1 |  | 1.70e-03 |
| Signaling events mediated by HDAC Class II | ESR1, HSP90AA1, NR3C1 |  | 1.83e-03 |
| IL2 signaling events mediated by PI3K | E2F1, HSP90AA1, PIK3R1 |  | 1.83e-03 |
| C-MYB transcription factor network | CDKN1A, CREBBP, EP300, SP1 |  | 2.12e-03 |
| amb2 Integrin signaling | FYN, LYN, SRC |  | 2.27e-03 |
| Internalization of ErbB1 | EGFR, PIK3R1, SRC |  | 2.27e-03 |
| Plasma membrane estrogen receptor signaling | ESR1, PIK3R1, SRC |  | 2.43e-03 |
| Integrin-linked kinase signaling | CREB1, HSP90AA1, JUN |  | 3.14e-03 |
| FOXA2 and FOXA3 transcription factor networks | CREB1, NR3C1, SP1 |  | 3.33e-03 |
| FoxO family signaling | CREBBP, EP300, USP7 |  | 3.95e-03 |
| Validated transcriptional targets of TAp63 isoforms | CDKN1A, EP300, SP1 |  | 5.13e-03 |
| RAC1 signaling pathway | JUN, STAT3, STAT5A |  | 5.13e-03 |
| Atypical NF-kappaB pathway | PIK3R1, SRC |  | 5.31e-03 |
| SHP2 signaling | EGFR, PIK3R1, STAT1 |  | 6.20e-03 |
| EPHA2 forward signaling | PIK3R1, SRC |  | 6.60e-03 |
| Hypoxic and oxygen homeostasis regulation of HIF-1-alpha | HSP90AA1, TP53 |  | 6.60e-03 |
| p38 signaling mediated by MAPKAP kinases | CREB1, TCF3 |  | 8.00e-03 |
| PDGFR-alpha signaling pathway | JUN, PIK3R1 |  | 8.75e-03 |
| BCR signaling pathway | JUN, LYN, PIK3R1 |  | 9.04e-03 |
| Signaling events mediated by PRL | CDKN1A, SRC |  | 9.52e-03 |
| CDC42 signaling events | JUN, PIK3R1, SRC |  | 9.76e-03 |
| p75(NTR)-mediated signaling | E2F1, PIK3R1, TP53 |  | 9.76e-03 |
| Signaling events mediated by HDAC Class I | CREBBP, EP300, STAT3 |  | 1.01e-02 |
| VEGFR3 signaling in lymphatic endothelium | CREB1, PIK3R1 |  | 1.11e-02 |
| S1P2 pathway | JUN, PIK3R1 |  | 1.20e-02 |
| Integrins in angiogenesis | HSP90AA1, PIK3R1, SRC |  | 1.29e-02 |
| TRAIL signaling pathway | PIK3R1, TRAF2 |  | 1.38e-02 |
| Signaling events mediated by Hepatocyte Growth Factor Receptor (c-Met) | JUN, PIK3R1, SRC |  | 1.38e-02 |
| Reelin signaling pathway | FYN, PIK3R1 |  | 1.47e-02 |
| VEGFR1 specific signals | HSP90AA1, PIK3R1 |  | 1.57e-02 |
| Nectin adhesion pathway | PIK3R1, SRC |  | 1.57e-02 |
| Osteopontin-mediated events | JUN, PIK3R1 |  | 1.66e-02 |
| Retinoic acid receptors-mediated signaling | CREBBP, EP300 |  | 1.66e-02 |
| Ephrin A reverse signaling | FYN |  | 1.97e-02 |
| Arf6 signaling events | EGFR, SRC |  | 2.18e-02 |
| IL1-mediated signaling events | JUN, PIK3R1 |  | 2.18e-02 |
| FAS (CD95) signaling pathway | PIK3R1, SRC |  | 2.52e-02 |
| EPHB forward signaling | PIK3R1, SRC |  | 2.64e-02 |
| E-cadherin signaling in the nascent adherens junction | PIK3R1, SRC |  | 2.64e-02 |
| Urokinase-type plasminogen activator (uPA) and uPAR-mediated signaling | EGFR, SRC |  | 2.87e-02 |
| CXCR3-mediated signaling events | PIK3R1, SRC |  | 3.12e-02 |
| a6b1 and a6b4 Integrin signaling | EGFR, PIK3R1 |  | 3.37e-02 |
| Presenilin action in Notch and Wnt signaling | CREBBP, JUN |  | 3.37e-02 |
| TNF receptor signaling pathway | STAT1, TRAF2 |  | 3.62e-02 |
| Validated transcriptional targets of deltaNp63 isoforms | TCF3, TCF7L1 |  | 3.62e-02 |
| Calcineurin-regulated NFAT-dependent transcription in lymphocytes | E2F1, JUN |  | 3.75e-02 |
| PLK3 signaling events | TP53 |  | 3.86e-02 |
| Ceramide signaling pathway | RB1, TRAF2 |  | 3.89e-02 |
| Caspase cascade in apoptosis | TRAF2, VIM |  | 4.15e-02 |
| Role of Calcineurin-dependent NFAT signaling in lymphocytes | CREBBP, EP300 |  | 4.98e-02 |
| Notch signaling pathway | CDKN1A, EP300 |  | 4.98e-02 |
| Neurotrophic factor-mediated Trk receptor signaling | PIK3R1, STAT3 |  | 5.56e-02 |
| EGFR-dependent Endothelin signaling events | EGFR |  | 5.69e-02 |
| Endothelins | JUN, SRC |  | 5.85e-02 |
| ALK2 signaling events | SMAD4 |  | 6.86e-02 |
| JNK signaling in the CD4+ TCR pathway | JUN |  | 8.56e-02 |
| LPA4-mediated signaling events | CREB1 |  | 9.66e-02 |
| Regulation of cytoplasmic and nuclear SMAD2/3 signaling | SMAD4 |  | 1.18e-01 |
| Signaling events mediated by the Hedgehog family | PIK3R1 |  | 1.33e-01 |
| Alpha9 beta1 integrin signaling events | SRC |  | 1.42e-01 |
| ALK1 signaling events | SMAD4 |  | 1.52e-01 |
| IL8- and CXCR1-mediated signaling events | LYN |  | 1.61e-01 |
| S1P3 pathway | SRC |  | 1.61e-01 |
| p38 MAPK signaling pathway | TRAF2 |  | 1.65e-01 |
| BARD1 signaling events | TP53 |  | 1.65e-01 |
| Calcium signaling in the CD4+ TCR pathway | JUN |  | 1.70e-01 |
| IGF1 pathway | PIK3R1 |  | 1.70e-01 |
| Aurora A signaling | TP53 |  | 1.74e-01 |
| Syndecan-2-mediated signaling events | SRC |  | 1.78e-01 |
| Alpha4 beta1 integrin signaling events | SRC |  | 1.83e-01 |
| IL8- and CXCR2-mediated signaling events | LYN |  | 1.87e-01 |
| HIV-1 Nef: Negative effector of Fas and TNF-alpha | TRAF2 |  | 1.87e-01 |
| Trk receptor signaling mediated by the MAPK pathway | CREB1 |  | 1.87e-01 |
| N-cadherin signaling events | PIK3R1 |  | 1.91e-01 |
| Aurora B signaling | VIM |  | 2.03e-01 |
| BMP receptor signaling | SMAD4 |  | 2.18e-01 |
| Stabilization and expansion of the E-cadherin adherens junction | EGFR |  | 2.21e-01 |
| Insulin Pathway | PIK3R1 |  | 2.25e-01 |
| PAR1-mediated thrombin signaling events | PIK3R1 |  | 2.25e-01 |
| RhoA signaling pathway | JUN |  | 2.28e-01 |
| Hedgehog signaling events mediated by Gli proteins | CREBBP |  | 2.35e-01 |
| TGF-beta receptor signaling | SMAD4 |  | 2.57e-01 |
| TCR signaling in na�ve CD8+ T cells | FYN |  | 2.60e-01 |
| Coregulation of Androgen receptor activity | AR |  | 2.80e-01 |
| TCR signaling in na�ve CD4+ T cells | FYN |  | 2.92e-01 |
| Downstream signaling in na�ve CD8+ T cells | JUN |  | 2.99e-01 |
