# Supplementary material for: A simple knowledge-based mining method for exploring hidden key molecules in a human biomolecular network
Source: BMC Syst Biol. 2012 Sep 15;6:124. doi: 10.1186/1752-0509-6-124 (PMC3740779; doi:10.1186/1752-0509-6-124)
Supplement: Additional file 2 — The collection of results for the Pathway Interaction Database analysis. The index.html file contains the links to the Pathway Interaction Database results for the various input genes. The input genes consist of the results of NetHiKe and Hubba (the top 30 genes of each). (Mini-websites, browse the index.html. [file 1752-0509-6-124-S2.zip › mini_web/Hubba_degree.html]

Batch query results : Pathway Interaction Database

- Jump to main content
- Jump to navigation

---

---

- Breadcrumb trail
  1. Home
  2. Batch query
  3. Batch query results

# Batch query results for NCI-Nature Curated data (Hubba degree)

| Pathway Name | Biomolecules in Group 1 | Biomolecules in Group 2 | P-value Help The pathways are ranked by the probability that they include biomolecules from the query list. The lower the p-value the greater the probability that the query list is biased towards a given pathway. The parameters for generating the p-value are the size of the query set, the number of biomolecules in a given pathway and the number of molecules in the database as a whole. |
| --- | --- | --- | --- |
| Regulation of nuclear SMAD2/3 signaling | AR, CREB1, CREBBP, EP300, ESR1, HDAC1, HDAC2, JUN, MYC, NR3C1, SIN3A, SIN3B, SMAD2, SMAD4, SP1 |  | 1.22e-18 |
| Glucocorticoid receptor regulatory network | CREB1, CREBBP, EP300, HDAC1, HDAC2, HSP90AA1, JUN, MAPK1, MDM2, NR3C1, STAT1, STAT5A, STAT5B, TBP, TP53 |  | 1.48e-18 |
| Regulation of Telomerase | E2F1, EGFR, ESR1, HDAC1, HDAC2, HSP90AA1, JUN, MAPK1, MYC, SIN3A, SIN3B, SP1 |  | 1.23e-14 |
| Signaling events mediated by TCPTP | CREBBP, EGFR, PIK3R1, SRC, STAT1, STAT3, STAT5A, STAT5B, STAT6 |  | 6.69e-12 |
| Notch-mediated HES/HEY network | AR, CREBBP, E2F1, EP300, HDAC1, JAK2, RB1, STAT3, YY1 |  | 2.36e-11 |
| PDGFR-beta signaling pathway | FYN, JAK2, JUN, LYN, MAPK1, MYC, PIK3R1, SRC, STAT1, STAT3, STAT5A, STAT5B |  | 3.02e-11 |
| Signaling events mediated by PTP1B | EGFR, FYN, JAK2, LYN, PIK3R1, SRC, STAT3, STAT5A, STAT5B |  | 4.16e-11 |
| Regulation of Androgen receptor activity | AR, CREBBP, EP300, HDAC1, HSP90AA1, JUN, MDM2, NR3C1, SRC |  | 4.99e-11 |
| GMCSF-mediated signaling events | JAK2, LYN, MAPK1, PIK3R1, STAT1, STAT3, STAT5A, STAT5B |  | 6.76e-11 |
| IL2-mediated signaling events | FYN, JUN, MAPK1, MYC, PIK3R1, STAT1, STAT3, STAT5A, STAT5B |  | 7.08e-11 |
| ErbB4 signaling events | FYN, JAK2, MAPK1, MDM2, PIK3R1, STAT5A, STAT5B |  | 4.88e-09 |
| IFN-gamma pathway | CREBBP, EP300, JAK2, MAPK1, PIK3R1, STAT1, STAT3 |  | 1.21e-08 |
| Regulation of retinoblastoma protein | CREBBP, E2F1, EP300, HDAC1, JUN, MDM2, RB1, TBP |  | 1.23e-08 |
| AP-1 transcription factor network | CREB1, EP300, ESR1, JUN, MYC, NR3C1, SP1, TP53 |  | 1.96e-08 |
| Signaling events mediated by HDAC Class I | CREBBP, EP300, HDAC1, HDAC2, SIN3A, SIN3B, STAT3, YY1 |  | 1.96e-08 |
| CXCR4-mediated signaling events | FYN, JAK2, LYN, PIK3R1, SRC, STAT1, STAT3, STAT5A, STAT5B |  | 2.16e-08 |
| Direct p53 effectors | CREBBP, E2F1, EGFR, EP300, HDAC2, JUN, MDM2, RB1, SP1, TP53 |  | 2.20e-08 |
| Validated targets of C-MYC transcriptional repression | CREB1, EP300, HDAC1, MYC, SMAD2, SMAD4, SP1, TBP |  | 2.45e-08 |
| IL5-mediated signaling events | JAK2, LYN, PIK3R1, STAT5A, STAT5B |  | 2.46e-08 |
| E2F transcription factor network | CREBBP, E2F1, EP300, HDAC1, MYC, RB1, SP1, YY1 |  | 3.38e-08 |
| Signaling events mediated by Stem cell factor receptor (c-Kit) | CREBBP, JAK2, LYN, PIK3R1, STAT1, STAT3, STAT5A |  | 6.24e-08 |
| EPO signaling pathway | JAK2, LYN, PIK3R1, STAT1, STAT5A, STAT5B |  | 1.08e-07 |
| EGF receptor (ErbB1) signaling pathway | EGFR, MAPK1, PIK3R1, SRC, STAT1, STAT3 |  | 1.08e-07 |
| Validated nuclear estrogen receptor alpha network | EP300, ESR1, HDAC1, JUN, MYC, SMAD4, STAT5A |  | 3.15e-07 |
| FOXM1 transcription factor network | CREBBP, EP300, ESR1, MYC, RB1, SP1 |  | 3.34e-07 |
| FOXA1 transcription factor network | AR, CREBBP, EP300, ESR1, JUN, SP1 |  | 4.44e-07 |
| ErbB2/ErbB3 signaling events | JAK2, JUN, MAPK1, PIK3R1, SRC, STAT3 |  | 4.44e-07 |
| ErbB1 downstream signaling | CREB1, EGFR, JUN, MAPK1, PIK3R1, SRC, STAT1, STAT3 |  | 6.08e-07 |
| IL6-mediated signaling events | JAK2, JUN, MYC, PIK3R1, STAT1, STAT3 |  | 6.64e-07 |
| IL3-mediated signaling events | HDAC1, JAK2, PIK3R1, STAT5A, STAT5B |  | 7.53e-07 |
| LKB1 signaling events | CREB1, ESR1, HSP90AA1, MYC, SMAD4, TP53 |  | 7.54e-07 |
| IL2 signaling events mediated by STAT5 | MYC, PIK3R1, SP1, STAT5A, STAT5B |  | 1.59e-06 |
| Nongenotropic Androgen signaling | AR, CREB1, MAPK1, PIK3R1, SRC |  | 1.89e-06 |
| FGF signaling pathway | JUN, MAPK1, PIK3R1, SRC, STAT1, STAT5B |  | 2.11e-06 |
| ATF-2 transcription factor network | CREB1, EP300, ESR1, JUN, MAPK1, RB1 |  | 2.59e-06 |
| p53 pathway | CREBBP, EP300, MDM2, TP53, USP7, YY1 |  | 2.87e-06 |
| IL4-mediated signaling events | JAK2, PIK3R1, SP1, STAT5A, STAT5B, STAT6 |  | 4.59e-06 |
| IL23-mediated signaling events | JAK2, PIK3R1, STAT1, STAT3, STAT5A |  | 4.66e-06 |
| HIF-1-alpha transcription factor network | CREB1, CREBBP, EP300, JUN, SMAD4, SP1 |  | 5.48e-06 |
| Signaling events regulated by Ret tyrosine kinase | CREB1, JUN, MAPK1, PIK3R1, SRC |  | 6.09e-06 |
| E-cadherin signaling in keratinocytes | EGFR, FYN, PIK3R1, SRC |  | 1.12e-05 |
| Presenilin action in Notch and Wnt signaling | CREBBP, HDAC1, JUN, MAPK1, MYC |  | 1.39e-05 |
| Regulation of nuclear beta catenin signaling and target gene transcription | AR, EP300, HDAC1, HDAC2, JUN, MYC |  | 1.64e-05 |
| Hedgehog signaling events mediated by Gli proteins | CREBBP, HDAC1, HDAC2, SIN3A, SIN3B |  | 1.71e-05 |
| Class I PI3K signaling events | FYN, HSP90AA1, LYN, PIK3R1, SRC |  | 1.90e-05 |
| Angiopoietin receptor Tie2-mediated signaling | FYN, MAPK1, PIK3R1, STAT5A, STAT5B |  | 1.90e-05 |
| Validated targets of C-MYC transcriptional activation | CREBBP, EP300, HSP90AA1, MYC, SMAD4, TP53 |  | 2.63e-05 |
| IL27-mediated signaling events | JAK2, STAT1, STAT3, STAT5A |  | 2.71e-05 |
| Glypican 1 network | FYN, LYN, SMAD2, SRC |  | 3.16e-05 |
| Ephrin B reverse signaling | FYN, LYN, PIK3R1, SRC |  | 4.84e-05 |
| Fc-epsilon receptor I signaling in mast cells | FYN, JUN, LYN, MAPK1, PIK3R1 |  | 5.50e-05 |
| Retinoic acid receptors-mediated signaling | CREBBP, EP300, HDAC1, MAPK1 |  | 5.52e-05 |
| Alpha-synuclein signaling | FYN, LYN, MAPK1, SRC |  | 6.27e-05 |
| Netrin-mediated signaling events | FYN, MAPK1, PIK3R1, SRC |  | 6.27e-05 |
| Signaling events mediated by focal adhesion kinase | FYN, JUN, MAPK1, PIK3R1, SRC |  | 6.41e-05 |
| LPA receptor mediated events | EGFR, JUN, LYN, PIK3R1, SRC |  | 7.44e-05 |
| IL12-mediated signaling events | JAK2, STAT1, STAT3, STAT5A, STAT6 |  | 8.60e-05 |
| Trk receptor signaling mediated by PI3K and PLC-gamma | CREB1, PIK3R1, SRC, STAT5A |  | 1.12e-04 |
| IL2 signaling events mediated by PI3K | E2F1, HSP90AA1, MYC, PIK3R1 |  | 1.24e-04 |
| Signaling mediated by p38-alpha and p38-beta | CREB1, ESR1, JUN, TP53 |  | 1.24e-04 |
| Signaling events mediated by VEGFR1 and VEGFR2 | FYN, HSP90AA1, MAPK1, PIK3R1, SRC |  | 1.29e-04 |
| p73 transcription factor network | EP300, MDM2, MYC, RB1, SP1 |  | 1.86e-04 |
| Syndecan-3-mediated signaling events | EGFR, FYN, SRC |  | 2.00e-04 |
| C-MYB transcription factor network | CREBBP, EP300, MYC, SIN3A, SP1 |  | 2.76e-04 |
| Posttranslational regulation of adherens junction stability and dissassembly | CREBBP, EGFR, FYN, SRC |  | 3.07e-04 |
| Regulation of cytoplasmic and nuclear SMAD2/3 signaling | MAPK1, SMAD2, SMAD4 |  | 3.29e-04 |
| Thromboxane A2 receptor signaling | EGFR, FYN, LYN, SRC |  | 5.50e-04 |
| Notch signaling pathway | EP300, HDAC1, MYC, YY1 |  | 6.27e-04 |
| VEGFR3 signaling in lymphatic endothelium | CREB1, MAPK1, PIK3R1 |  | 6.42e-04 |
| SHP2 signaling | EGFR, JAK2, PIK3R1, STAT1 |  | 6.69e-04 |
| S1P2 pathway | JUN, MAPK1, PIK3R1 |  | 7.21e-04 |
| Endothelins | JAK2, JUN, MAPK1, SRC |  | 9.04e-04 |
| S1P3 pathway | JAK2, MAPK1, SRC |  | 9.94e-04 |
| VEGFR1 specific signals | HSP90AA1, MAPK1, PIK3R1 |  | 1.10e-03 |
| BCR signaling pathway | JUN, LYN, MAPK1, PIK3R1 |  | 1.13e-03 |
| Osteopontin-mediated events | JUN, MAPK1, PIK3R1 |  | 1.21e-03 |
| CD40/CD40L signaling | JUN, MYC, STAT5A |  | 1.21e-03 |
| Regulation of p38-alpha and p38-beta | FYN, LYN, SRC |  | 1.21e-03 |
| Nephrin/Neph1 signaling in the kidney podocyte | FYN, JUN, PIK3R1 |  | 1.21e-03 |
| CDC42 signaling events | JUN, MAPK1, PIK3R1, SRC |  | 1.26e-03 |
| EPHA forward signaling | FYN, LYN, SRC |  | 1.58e-03 |
| IL12 signaling mediated by STAT4 | CREBBP, JUN, STAT3 |  | 1.71e-03 |
| Integrins in angiogenesis | HSP90AA1, MAPK1, PIK3R1, SRC |  | 1.86e-03 |
| HIF-2-alpha transcription factor network | CREBBP, EP300, SP1 |  | 1.86e-03 |
| Validated transcriptional targets of AP1 family members Fra1 and Fra2 | EP300, JUN, SP1 |  | 2.01e-03 |
| Signaling events mediated by Hepatocyte Growth Factor Receptor (c-Met) | JUN, MAPK1, PIK3R1, SRC |  | 2.03e-03 |
| Signaling events mediated by HDAC Class II | ESR1, HSP90AA1, NR3C1 |  | 2.16e-03 |
| Signaling events mediated by HDAC Class III | CREBBP, EP300, TP53 |  | 2.33e-03 |
| EPHB forward signaling | MAPK1, PIK3R1, SRC |  | 2.50e-03 |
| amb2 Integrin signaling | FYN, LYN, SRC |  | 2.68e-03 |
| Internalization of ErbB1 | EGFR, PIK3R1, SRC |  | 2.68e-03 |
| Plasma membrane estrogen receptor signaling | ESR1, PIK3R1, SRC |  | 2.87e-03 |
| CXCR3-mediated signaling events | MAPK1, PIK3R1, SRC |  | 3.27e-03 |
| Integrin-linked kinase signaling | CREB1, HSP90AA1, JUN |  | 3.70e-03 |
| FOXA2 and FOXA3 transcription factor networks | CREB1, NR3C1, SP1 |  | 3.92e-03 |
| FoxO family signaling | CREBBP, EP300, USP7 |  | 4.65e-03 |
| Ceramide signaling pathway | MAPK1, MYC, RB1 |  | 4.65e-03 |
| Sumoylation by RanBP2 regulates transcriptional repression | HDAC1, MDM2 |  | 4.66e-03 |
| ErbB receptor signaling network | EGFR, HSP90AA1 |  | 5.29e-03 |
| Atypical NF-kappaB pathway | PIK3R1, SRC |  | 5.95e-03 |
| Validated transcriptional targets of TAp63 isoforms | EP300, MDM2, SP1 |  | 6.02e-03 |
| RAC1 signaling pathway | JUN, STAT3, STAT5A |  | 6.02e-03 |
| EPHA2 forward signaling | PIK3R1, SRC |  | 7.38e-03 |
| Hypoxic and oxygen homeostasis regulation of HIF-1-alpha | HSP90AA1, TP53 |  | 7.38e-03 |
| Neurotrophic factor-mediated Trk receptor signaling | MAPK1, PIK3R1, STAT3 |  | 8.29e-03 |
| PDGFR-alpha signaling pathway | JUN, PIK3R1 |  | 9.78e-03 |
| Signaling events mediated by PRL | MAPK1, SRC |  | 1.06e-02 |
| p75(NTR)-mediated signaling | E2F1, PIK3R1, TP53 |  | 1.14e-02 |
| ALK1 signaling events | MAPK1, SMAD4 |  | 1.44e-02 |
| TRAIL signaling pathway | MAPK1, PIK3R1 |  | 1.54e-02 |
| Reelin signaling pathway | FYN, PIK3R1 |  | 1.64e-02 |
| Nectin adhesion pathway | PIK3R1, SRC |  | 1.74e-02 |
| Aurora A signaling | MDM2, TP53 |  | 1.96e-02 |
| Syndecan-2-mediated signaling events | MAPK1, SRC |  | 2.08e-02 |
| Ephrin A reverse signaling | FYN |  | 2.09e-02 |
| Trk receptor signaling mediated by the MAPK pathway | CREB1, MAPK1 |  | 2.31e-02 |
| Class I PI3K signaling events mediated by Akt | HSP90AA1, SRC |  | 2.43e-02 |
| Arf6 signaling events | EGFR, SRC |  | 2.43e-02 |
| IL1-mediated signaling events | JUN, PIK3R1 |  | 2.43e-02 |
| FAS (CD95) signaling pathway | PIK3R1, SRC |  | 2.80e-02 |
| E-cadherin signaling in the nascent adherens junction | PIK3R1, SRC |  | 2.93e-02 |
| Urokinase-type plasminogen activator (uPA) and uPAR-mediated signaling | EGFR, SRC |  | 3.19e-02 |
| BMP receptor signaling | MAPK1, SMAD4 |  | 3.32e-02 |
| a6b1 and a6b4 Integrin signaling | EGFR, PIK3R1 |  | 3.73e-02 |
| PLK3 signaling events | TP53 |  | 4.09e-02 |
| Class IB PI3K non-lipid kinase events | MAPK1 |  | 4.09e-02 |
| Calcineurin-regulated NFAT-dependent transcription in lymphocytes | E2F1, JUN |  | 4.15e-02 |
| TGF-beta receptor signaling | SMAD2, SMAD4 |  | 5.03e-02 |
| Role of Calcineurin-dependent NFAT signaling in lymphocytes | CREBBP, EP300 |  | 5.49e-02 |
| EGFR-dependent Endothelin signaling events | EGFR |  | 6.01e-02 |
| ALK2 signaling events | SMAD4 |  | 7.25e-02 |
| mTOR signaling pathway | MAPK1, YY1 |  | 7.40e-02 |
| Downstream signaling in na�ve CD8+ T cells | JUN, MAPK1 |  | 7.56e-02 |
| JNK signaling in the CD4+ TCR pathway | JUN |  | 9.03e-02 |
| S1P4 pathway | MAPK1 |  | 9.03e-02 |
| Ras signaling in the CD4+ TCR pathway | MAPK1 |  | 9.03e-02 |
| Arf6 downstream pathway | MAPK1 |  | 9.61e-02 |
| LPA4-mediated signaling events | CREB1 |  | 1.02e-01 |
| S1P1 pathway | MAPK1 |  | 1.29e-01 |
| p38 signaling mediated by MAPKAP kinases | CREB1 |  | 1.29e-01 |
| Cellular roles of Anthrax toxin | MAPK1 |  | 1.34e-01 |
| Signaling events mediated by the Hedgehog family | PIK3R1 |  | 1.39e-01 |
| Alpha9 beta1 integrin signaling events | SRC |  | 1.50e-01 |
| C-MYC pathway | MYC |  | 1.50e-01 |
| IL8- and CXCR1-mediated signaling events | LYN |  | 1.69e-01 |
| BARD1 signaling events | TP53 |  | 1.73e-01 |
| Calcium signaling in the CD4+ TCR pathway | JUN |  | 1.78e-01 |
| IGF1 pathway | PIK3R1 |  | 1.78e-01 |
| Alpha4 beta1 integrin signaling events | SRC |  | 1.91e-01 |
| ATM pathway | MDM2 |  | 1.91e-01 |
| IL8- and CXCR2-mediated signaling events | LYN |  | 1.95e-01 |
| N-cadherin signaling events | PIK3R1 |  | 2.00e-01 |
| ATR signaling pathway | MDM2 |  | 2.16e-01 |
| Stabilization and expansion of the E-cadherin adherens junction | EGFR |  | 2.31e-01 |
| Insulin Pathway | PIK3R1 |  | 2.34e-01 |
| PAR1-mediated thrombin signaling events | PIK3R1 |  | 2.34e-01 |
| RhoA signaling pathway | JUN |  | 2.38e-01 |
| TNF receptor signaling pathway | STAT1 |  | 2.45e-01 |
| Validated transcriptional targets of deltaNp63 isoforms | MDM2 |  | 2.45e-01 |
| Syndecan-1-mediated signaling events | MAPK1 |  | 2.48e-01 |
| TCR signaling in na�ve CD8+ T cells | FYN |  | 2.70e-01 |
| Coregulation of Androgen receptor activity | AR |  | 2.89e-01 |
| TCR signaling in na�ve CD4+ T cells | FYN |  | 3.02e-01 |
