# Supplementary material for: A simple knowledge-based mining method for exploring hidden key molecules in a human biomolecular network
Source: BMC Syst Biol. 2012 Sep 15;6:124. doi: 10.1186/1752-0509-6-124 (PMC3740779; doi:10.1186/1752-0509-6-124)
Supplement: Additional file 2 — The collection of results for the Pathway Interaction Database analysis. The index.html file contains the links to the Pathway Interaction Database results for the various input genes. The input genes consist of the results of NetHiKe and Hubba (the top 30 genes of each). (Mini-websites, browse the index.html. [file 1752-0509-6-124-S2.zip › mini_web/Hubba_DMNC.html]

Batch query results : Pathway Interaction Database

- Jump to main content
- Jump to navigation

---

---

- Breadcrumb trail
  1. Home
  2. Batch query
  3. Batch query results

# Batch query results for NCI-Nature Curated data (Hubba DMNC)

| Pathway Name | Biomolecules in Group 1 | Biomolecules in Group 2 | P-value Help The pathways are ranked by the probability that they include biomolecules from the query list. The lower the p-value the greater the probability that the query list is biased towards a given pathway. The parameters for generating the p-value are the size of the query set, the number of biomolecules in a given pathway and the number of molecules in the database as a whole. |
| --- | --- | --- | --- |
| Regulation of nuclear SMAD2/3 signaling | CDK2, E2F4, E2F5, FOS, FOXO4, KAT2B, NCOA1, NCOA2, NR3C1, SP1, VDR |  | 1.78e-12 |
| Glucocorticoid receptor regulatory network | FOS, NCOA1, NCOA2, NR3C1, RELA, STAT1, STAT5A, STAT5B, TBP |  | 1.78e-09 |
| Signaling events mediated by TCPTP | EGF, PTPN1, STAT1, STAT5A, STAT5B, STAT6 |  | 2.67e-07 |
| E2F transcription factor network | CDK2, CEBPA, E2F4, E2F5, KAT2B, RBL2, SP1 |  | 4.44e-07 |
| FoxO family signaling | CDK2, CTNNB1, FOXO4, KAT2B, RBL2, SKP2 |  | 6.70e-07 |
| IL2-mediated signaling events | CDK2, FOS, PTPN11, STAT1, STAT5A, STAT5B |  | 1.19e-06 |
| Retinoic acid receptors-mediated signaling | KAT2B, NCOA1, NCOA2, NRIP1, VDR |  | 1.39e-06 |
| EPO signaling pathway | PTPN11, PTPN6, STAT1, STAT5A, STAT5B |  | 2.60e-06 |
| EGF receptor (ErbB1) signaling pathway | EGF, PTPN1, PTPN11, PTPN6, STAT1 |  | 2.60e-06 |
| GMCSF-mediated signaling events | FOS, PTPN11, STAT1, STAT5A, STAT5B |  | 3.00e-06 |
| CXCR4-mediated signaling events | DNM1, PTPN11, PTPN6, STAT1, STAT2, STAT5A, STAT5B |  | 3.48e-06 |
| IL12-mediated signaling events | FOS, RELA, STAT1, STAT4, STAT5A, STAT6 |  | 3.83e-06 |
| Regulation of Androgen receptor activity | CEBPA, KAT2B, NCOA1, NCOA2, NR3C1 |  | 2.08e-05 |
| IL27-mediated signaling events | STAT1, STAT2, STAT4, STAT5A |  | 2.13e-05 |
| IL2 signaling events mediated by STAT5 | PTPN11, SP1, STAT5A, STAT5B |  | 3.82e-05 |
| Coregulation of Androgen receptor activity | CTNNB1, MED1, NCOA2, NRIP1, PELP1 |  | 4.80e-05 |
| IL4-mediated signaling events | PTPN6, SP1, STAT5A, STAT5B, STAT6 |  | 5.58e-05 |
| Regulation of retinoblastoma protein | CDK2, CEBPA, E2F4, SKP2, TBP |  | 6.45e-05 |
| Validated nuclear estrogen receptor alpha network | MED1, NCOA1, NCOA2, NRIP1, STAT5A |  | 6.92e-05 |
| IL23-mediated signaling events | RELA, STAT1, STAT4, STAT5A |  | 8.83e-05 |
| IL5-mediated signaling events | PTPN11, STAT5A, STAT5B |  | 9.14e-05 |
| ErbB receptor signaling network | EGF, ERBB3, ERBB4 |  | 1.39e-04 |
| FOXM1 transcription factor network | CDK2, FOS, SKP2, SP1 |  | 1.45e-04 |
| PDGFR-beta signaling pathway | FOS, PTPN1, PTPN11, STAT1, STAT5A, STAT5B |  | 1.61e-04 |
| Posttranslational regulation of adherens junction stability and dissassembly | CTNNB1, EGF, PTPN1, PTPN6 |  | 2.44e-04 |
| Angiopoietin receptor Tie2-mediated signaling | PTPN11, RELA, STAT5A, STAT5B |  | 2.64e-04 |
| Signaling events mediated by PTP1B | EGF, PTPN1, STAT5A, STAT5B |  | 3.31e-04 |
| Signaling events mediated by Stem cell factor receptor (c-Kit) | PTPN11, PTPN6, STAT1, STAT5A |  | 3.83e-04 |
| FGF signaling pathway | FOS, PTPN11, STAT1, STAT5B |  | 4.69e-04 |
| IL3-mediated signaling events | PTPN11, STAT5A, STAT5B |  | 6.07e-04 |
| RXR and RAR heterodimerization with other nuclear receptor | MED1, NCOA1, VDR |  | 6.78e-04 |
| HIF-1-alpha transcription factor network | FOS, NCOA1, NCOA2, SP1 |  | 8.58e-04 |
| AP-1 transcription factor network | CTNNB1, FOS, NR3C1, SP1 |  | 1.06e-03 |
| N-cadherin signaling events | CTNNB1, PTPN1, PTPN11 |  | 1.57e-03 |
| ErbB4 signaling events | ERBB4, STAT5A, STAT5B |  | 1.83e-03 |
| ErbB2/ErbB3 signaling events | ERBB3, FOS, PTPN11 |  | 2.77e-03 |
| FOXA1 transcription factor network | FOS, NRIP1, SP1 |  | 2.77e-03 |
| FOXA2 and FOXA3 transcription factor networks | CEBPA, NR3C1, SP1 |  | 3.33e-03 |
| IL6-mediated signaling events | FOS, PTPN11, STAT1 |  | 3.33e-03 |
| Validated nuclear estrogen receptor beta network | NCOA1, NCOA2 |  | 4.15e-03 |
| SHP2 signaling | EGF, PTPN11, STAT1 |  | 6.20e-03 |
| p53 pathway | CDK2, KAT2B, SKP2 |  | 6.49e-03 |
| Fc-epsilon receptor I signaling in mast cells | FOS, PTPN11, RELA |  | 6.78e-03 |
| BCR signaling pathway | FOS, PTPN6, RELA |  | 9.04e-03 |
| Regulation of Telomerase | EGF, FOS, SP1 |  | 9.40e-03 |
| Validated targets of C-MYC transcriptional repression | CEBPA, SP1, TBP |  | 1.09e-02 |
| Signaling events mediated by VEGFR1 and VEGFR2 | CTNNB1, PTPN11, PTPN6 |  | 1.09e-02 |
| p73 transcription factor network | CDK2, RELA, SP1 |  | 1.33e-02 |
| Signaling events mediated by Hepatocyte Growth Factor Receptor (c-Met) | CTNNB1, PTPN1, PTPN11 |  | 1.38e-02 |
| IGF1 pathway | PTPN1, PTPN11 |  | 1.66e-02 |
| Osteopontin-mediated events | FOS, RELA |  | 1.66e-02 |
| CD40/CD40L signaling | RELA, STAT5A |  | 1.66e-02 |
| Nongenotropic Androgen signaling | FOS, PELP1 |  | 1.66e-02 |
| E-cadherin signaling events | CTNNB1 |  | 1.97e-02 |
| IL12 signaling mediated by STAT4 | FOS, STAT4 |  | 2.08e-02 |
| IL2 signaling events mediated by PI3K | PTPN11, RELA |  | 2.41e-02 |
| Signaling events mediated by HDAC Class III | FOXO4, KAT2B |  | 2.52e-02 |
| Internalization of ErbB1 | DNM1, EGF |  | 2.75e-02 |
| IFN-gamma pathway | PTPN11, STAT1 |  | 2.99e-02 |
| ErbB1 downstream signaling | EGF, FOS, STAT1 |  | 3.00e-02 |
| Stabilization and expansion of the E-cadherin adherens junction | CTNNB1, EGF |  | 3.12e-02 |
| Insulin Pathway | PTPN1, PTPN11 |  | 3.24e-02 |
| a6b1 and a6b4 Integrin signaling | EGF, ERBB3 |  | 3.37e-02 |
| Presenilin action in Notch and Wnt signaling | CTNNB1, FOS |  | 3.37e-02 |
| TNF receptor signaling pathway | RELA, STAT1 |  | 3.62e-02 |
| Calcineurin-regulated NFAT-dependent transcription in lymphocytes | FOS, PTPN1 |  | 3.75e-02 |
| Ceramide signaling pathway | EGF, RELA |  | 3.89e-02 |
| Validated transcriptional targets of TAp63 isoforms | SP1, VDR |  | 4.56e-02 |
| RAC1 signaling pathway | CTNNB1, STAT5A |  | 4.56e-02 |
| Thromboxane A2 receptor signaling | DNM1, EGF |  | 4.70e-02 |
| Notch signaling pathway | DNM1, SKP2 |  | 4.98e-02 |
| Neurotrophic factor-mediated Trk receptor signaling | DNM1, PTPN11 |  | 5.56e-02 |
| EGFR-dependent Endothelin signaling events | EGF |  | 5.69e-02 |
| LPA receptor mediated events | FOS, RELA |  | 6.00e-02 |
| TCR signaling in na�ve CD4+ T cells | PTPN11, PTPN6 |  | 6.45e-02 |
| Signaling events mediated by HDAC Class I | KAT2B, RELA |  | 6.90e-02 |
| Downstream signaling in na�ve CD8+ T cells | FOS, STAT4 |  | 6.90e-02 |
| Regulation of nuclear beta catenin signaling and target gene transcription | CTNNB1, NCOA2 |  | 8.44e-02 |
| Ras signaling in the CD4+ TCR pathway | FOS |  | 8.56e-02 |
| C-MYB transcription factor network | CEBPA, SP1 |  | 9.22e-02 |
| Atypical NF-kappaB pathway | RELA |  | 1.02e-01 |
| Degradation of beta catenin | CTNNB1 |  | 1.07e-01 |
| Canonical Wnt signaling pathway | CTNNB1 |  | 1.23e-01 |
| E-cadherin signaling in keratinocytes | CTNNB1 |  | 1.23e-01 |
| PDGFR-alpha signaling pathway | FOS |  | 1.28e-01 |
| Canonical NF-kappaB pathway | RELA |  | 1.33e-01 |
| Signaling events mediated by PRL | CDK2 |  | 1.33e-01 |
| C-MYC pathway | SKP2 |  | 1.42e-01 |
| S1P2 pathway | FOS |  | 1.47e-01 |
| IL8- and CXCR1-mediated signaling events | DNM1 |  | 1.61e-01 |
| VEGFR1 specific signals | PTPN11 |  | 1.65e-01 |
| Nectin adhesion pathway | CTNNB1 |  | 1.65e-01 |
| BARD1 signaling events | CDK2 |  | 1.65e-01 |
| Ephrin B reverse signaling | DNM1 |  | 1.65e-01 |
| Calcium signaling in the CD4+ TCR pathway | FOS |  | 1.70e-01 |
| Direct p53 effectors | SP1, VDR |  | 1.74e-01 |
| IL8- and CXCR2-mediated signaling events | DNM1 |  | 1.87e-01 |
| HIV-1 Nef: Negative effector of Fas and TNF-alpha | RELA |  | 1.87e-01 |
| Trk receptor signaling mediated by the MAPK pathway | FOS |  | 1.87e-01 |
| HIF-2-alpha transcription factor network | SP1 |  | 1.91e-01 |
| Arf6 signaling events | EGF |  | 1.91e-01 |
| IL1-mediated signaling events | RELA |  | 1.91e-01 |
| Class I PI3K signaling events mediated by Akt | FOXO4 |  | 1.91e-01 |
| Trk receptor signaling mediated by PI3K and PLC-gamma | STAT5A |  | 1.95e-01 |
| Validated transcriptional targets of AP1 family members Fra1 and Fra2 | SP1 |  | 1.95e-01 |
| Signaling events mediated by HDAC Class II | NR3C1 |  | 1.99e-01 |
| Signaling events regulated by Ret tyrosine kinase | PTPN11 |  | 2.03e-01 |
| EPHB forward signaling | DNM1 |  | 2.07e-01 |
| E-cadherin signaling in the nascent adherens junction | CTNNB1 |  | 2.07e-01 |
| ATR signaling pathway | CDK2 |  | 2.07e-01 |
| Plasma membrane estrogen receptor signaling | PELP1 |  | 2.14e-01 |
| CXCR3-mediated signaling events | DNM1 |  | 2.21e-01 |
| PAR1-mediated thrombin signaling events | DNM1 |  | 2.25e-01 |
| Integrin-linked kinase signaling | CTNNB1 |  | 2.28e-01 |
| RhoA signaling pathway | FOS |  | 2.28e-01 |
| Validated transcriptional targets of deltaNp63 isoforms | VDR |  | 2.35e-01 |
| Arf6 trafficking events | CTNNB1 |  | 2.39e-01 |
| Notch-mediated HES/HEY network | NCOA1 |  | 2.39e-01 |
| TGF-beta receptor signaling | CTNNB1 |  | 2.57e-01 |
| TCR signaling in na�ve CD8+ T cells | PTPN6 |  | 2.60e-01 |
| ATF-2 transcription factor network | FOS |  | 2.69e-01 |
| Endothelins | FOS |  | 2.82e-01 |
| mTOR signaling pathway | CDK2 |  | 2.97e-01 |
| CDC42 signaling events | CTNNB1 |  | 2.97e-01 |
| Integrins in angiogenesis | PTPN11 |  | 3.14e-01 |
