# Supplementary material for: A simple knowledge-based mining method for exploring hidden key molecules in a human biomolecular network
Source: BMC Syst Biol. 2012 Sep 15;6:124. doi: 10.1186/1752-0509-6-124 (PMC3740779; doi:10.1186/1752-0509-6-124)
Supplement: Additional file 2 — The collection of results for the Pathway Interaction Database analysis. The index.html file contains the links to the Pathway Interaction Database results for the various input genes. The input genes consist of the results of NetHiKe and Hubba (the top 30 genes of each). (Mini-websites, browse the index.html. [file 1752-0509-6-124-S2.zip › mini_web/Hubba_EPC.html]

Batch query results : Pathway Interaction Database

- Jump to main content
- Jump to navigation

---

---

- Breadcrumb trail
  1. Home
  2. Batch query
  3. Batch query results

# Batch query results for NCI-Nature Curated data (Hubba EPC)

| Pathway Name | Biomolecules in Group 1 | Biomolecules in Group 2 | P-value Help The pathways are ranked by the probability that they include biomolecules from the query list. The lower the p-value the greater the probability that the query list is biased towards a given pathway. The parameters for generating the p-value are the size of the query set, the number of biomolecules in a given pathway and the number of molecules in the database as a whole. |
| --- | --- | --- | --- |
| Regulation of nuclear SMAD2/3 signaling | AKT1, AR, CEBPB, CREB1, CREBBP, EP300, ESR1, HDAC1, HDAC2, JUN, MYC, NCOR1, NR3C1, SMAD2, SMAD4, SP1 |  | 3.72e-20 |
| Glucocorticoid receptor regulatory network | AKT1, CREB1, CREBBP, EP300, HDAC1, HDAC2, HSP90AA1, JUN, MAPK1, MDM2, NR3C1, STAT1, STAT5A, STAT5B, TBP, TP53 |  | 4.59e-20 |
| Validated nuclear estrogen receptor alpha network | CEBPB, EP300, ESR1, HDAC1, HDAC4, JUN, MYC, NCOR1, NCOR2, SMAD4, STAT5A |  | 6.22e-13 |
| Notch-mediated HES/HEY network | AR, CREBBP, E2F1, EP300, HDAC1, JAK2, NCOR1, NCOR2, RB1, STAT3 |  | 6.91e-13 |
| Regulation of Telomerase | AKT1, E2F1, EGFR, ESR1, HDAC1, HDAC2, HSP90AA1, JUN, MAPK1, MYC, SP1 |  | 7.36e-13 |
| Signaling events mediated by PTP1B | AKT1, EGFR, FYN, JAK2, LYN, SRC, STAT3, STAT5A, STAT5B |  | 5.50e-11 |
| Regulation of Androgen receptor activity | AR, CREBBP, EP300, HDAC1, HSP90AA1, JUN, MDM2, NR3C1, SRC |  | 6.59e-11 |
| Signaling events mediated by TCPTP | CREBBP, EGFR, SRC, STAT1, STAT3, STAT5A, STAT5B, STAT6 |  | 3.98e-10 |
| IFN-gamma pathway | AKT1, CEBPB, CREBBP, EP300, JAK2, MAPK1, STAT1, STAT3 |  | 3.98e-10 |
| Regulation of retinoblastoma protein | CEBPB, CREBBP, E2F1, EP300, HDAC1, JUN, MDM2, RB1, TBP |  | 5.89e-10 |
| PDGFR-beta signaling pathway | FYN, JAK2, JUN, LYN, MAPK1, MYC, SRC, STAT1, STAT3, STAT5A, STAT5B |  | 8.57e-10 |
| AP-1 transcription factor network | CREB1, CTNNB1, EP300, ESR1, JUN, MYC, NR3C1, SP1, TP53 |  | 1.00e-09 |
| IL2-mediated signaling events | FYN, JUN, MAPK1, MYC, STAT1, STAT3, STAT5A, STAT5B |  | 3.12e-09 |
| GMCSF-mediated signaling events | JAK2, LYN, MAPK1, STAT1, STAT3, STAT5A, STAT5B |  | 4.03e-09 |
| ErbB4 signaling events | FYN, JAK2, MAPK1, MDM2, NCOR1, STAT5A, STAT5B |  | 6.01e-09 |
| FOXA1 transcription factor network | AR, CEBPB, CREBBP, EP300, ESR1, JUN, SP1 |  | 1.76e-08 |
| Signaling events mediated by HDAC Class I | CREBBP, EP300, HDAC1, HDAC2, HDAC4, NCOR1, NCOR2, STAT3 |  | 2.49e-08 |
| CXCR4-mediated signaling events | AKT1, FYN, JAK2, LYN, SRC, STAT1, STAT3, STAT5A, STAT5B |  | 2.83e-08 |
| IL6-mediated signaling events | AKT1, CEBPB, JAK2, JUN, MYC, STAT1, STAT3 |  | 2.84e-08 |
| Direct p53 effectors | CREBBP, E2F1, EGFR, EP300, HDAC2, JUN, MDM2, RB1, SP1, TP53 |  | 2.96e-08 |
| Validated targets of C-MYC transcriptional repression | CREB1, EP300, HDAC1, MYC, SMAD2, SMAD4, SP1, TBP |  | 3.11e-08 |
| Retinoic acid receptors-mediated signaling | AKT1, CREBBP, EP300, HDAC1, MAPK1, NCOR2 |  | 5.97e-08 |
| Signaling events mediated by Stem cell factor receptor (c-Kit) | AKT1, CREBBP, JAK2, LYN, STAT1, STAT3, STAT5A |  | 7.68e-08 |
| E-cadherin signaling in keratinocytes | AKT1, CTNNB1, EGFR, FYN, SRC |  | 2.78e-07 |
| IL4-mediated signaling events | AKT1, CEBPB, JAK2, SP1, STAT5A, STAT5B, STAT6 |  | 2.83e-07 |
| HIF-1-alpha transcription factor network | AKT1, CREB1, CREBBP, EP300, JUN, SMAD4, SP1 |  | 3.49e-07 |
| FOXM1 transcription factor network | CREBBP, EP300, ESR1, MYC, RB1, SP1 |  | 3.98e-07 |
| ErbB2/ErbB3 signaling events | AKT1, JAK2, JUN, MAPK1, SRC, STAT3 |  | 5.29e-07 |
| Presenilin action in Notch and Wnt signaling | CREBBP, CTNNB1, HDAC1, JUN, MAPK1, MYC |  | 6.93e-07 |
| ErbB1 downstream signaling | AKT1, CREB1, EGFR, JUN, MAPK1, SRC, STAT1, STAT3 |  | 7.65e-07 |
| E2F transcription factor network | CREBBP, E2F1, EP300, HDAC1, MYC, RB1, SP1 |  | 8.32e-07 |
| IL3-mediated signaling events | CEBPB, HDAC1, JAK2, STAT5A, STAT5B |  | 8.70e-07 |
| LKB1 signaling events | CREB1, ESR1, HSP90AA1, MYC, SMAD4, TP53 |  | 8.97e-07 |
| Regulation of nuclear beta catenin signaling and target gene transcription | AR, CTNNB1, EP300, HDAC1, HDAC2, JUN, MYC |  | 1.28e-06 |
| Nongenotropic Androgen signaling | AKT1, AR, CREB1, MAPK1, SRC |  | 2.18e-06 |
| IL5-mediated signaling events | JAK2, LYN, STAT5A, STAT5B |  | 2.19e-06 |
| FGF signaling pathway | AKT1, JUN, MAPK1, SRC, STAT1, STAT5B |  | 2.51e-06 |
| ATF-2 transcription factor network | CREB1, EP300, ESR1, JUN, MAPK1, RB1 |  | 3.08e-06 |
| EPO signaling pathway | JAK2, LYN, STAT1, STAT5A, STAT5B |  | 4.06e-06 |
| EGF receptor (ErbB1) signaling pathway | EGFR, MAPK1, SRC, STAT1, STAT3 |  | 4.06e-06 |
| Signaling events mediated by HDAC Class II | ESR1, HDAC4, HSP90AA1, NCOR2, NR3C1 |  | 6.16e-06 |
| Signaling mediated by p38-alpha and p38-beta | CEBPB, CREB1, ESR1, JUN, TP53 |  | 6.16e-06 |
| Signaling events mediated by VEGFR1 and VEGFR2 | AKT1, CTNNB1, FYN, HSP90AA1, MAPK1, SRC |  | 1.07e-05 |
| Integrin-linked kinase signaling | AKT1, CREB1, CTNNB1, HSP90AA1, JUN |  | 1.60e-05 |
| FOXA2 and FOXA3 transcription factor networks | AKT1, CEBPB, CREB1, NR3C1, SP1 |  | 1.78e-05 |
| Posttranslational regulation of adherens junction stability and dissassembly | CREBBP, CTNNB1, EGFR, FYN, SRC |  | 1.97e-05 |
| Angiopoietin receptor Tie2-mediated signaling | AKT1, FYN, MAPK1, STAT5A, STAT5B |  | 2.18e-05 |
| C-MYB transcription factor network | CEBPB, CREBBP, EP300, MYC, NCOR1, SP1 |  | 2.72e-05 |
| IL27-mediated signaling events | JAK2, STAT1, STAT3, STAT5A |  | 3.03e-05 |
| Validated targets of C-MYC transcriptional activation | CREBBP, EP300, HSP90AA1, MYC, SMAD4, TP53 |  | 3.10e-05 |
| Glypican 1 network | FYN, LYN, SMAD2, SRC |  | 3.54e-05 |
| Thromboxane A2 receptor signaling | AKT1, EGFR, FYN, LYN, SRC |  | 4.18e-05 |
| S1P3 pathway | AKT1, JAK2, MAPK1, SRC |  | 4.72e-05 |
| Notch signaling pathway | EP300, HDAC1, MYC, NCOR1, NCOR2 |  | 4.95e-05 |
| IL2 signaling events mediated by STAT5 | MYC, SP1, STAT5A, STAT5B |  | 5.41e-05 |
| p53 pathway | AKT1, CREBBP, EP300, MDM2, TP53 |  | 5.83e-05 |
| CD40/CD40L signaling | AKT1, JUN, MYC, STAT5A |  | 6.17e-05 |
| Fc-epsilon receptor I signaling in mast cells | AKT1, FYN, JUN, LYN, MAPK1 |  | 6.31e-05 |
| Alpha-synuclein signaling | FYN, LYN, MAPK1, SRC |  | 7.01e-05 |
| Endothelins | AKT1, JAK2, JUN, MAPK1, SRC |  | 7.93e-05 |
| LPA receptor mediated events | AKT1, EGFR, JUN, LYN, SRC |  | 8.54e-05 |
| IL12-mediated signaling events | JAK2, STAT1, STAT3, STAT5A, STAT6 |  | 9.86e-05 |
| Trk receptor signaling mediated by PI3K and PLC-gamma | AKT1, CREB1, SRC, STAT5A |  | 1.25e-04 |
| IL23-mediated signaling events | JAK2, STAT1, STAT3, STAT5A |  | 1.25e-04 |
| IL2 signaling events mediated by PI3K | AKT1, E2F1, HSP90AA1, MYC |  | 1.38e-04 |
| Sumoylation by RanBP2 regulates transcriptional repression | HDAC1, HDAC4, MDM2 |  | 1.48e-04 |
| Signaling events mediated by HDAC Class III | CREBBP, EP300, HDAC4, TP53 |  | 1.53e-04 |
| Signaling events regulated by Ret tyrosine kinase | CREB1, JUN, MAPK1, SRC |  | 1.53e-04 |
| amb2 Integrin signaling | AKT1, FYN, LYN, SRC |  | 1.86e-04 |
| p73 transcription factor network | EP300, MDM2, MYC, RB1, SP1 |  | 2.13e-04 |
| Syndecan-3-mediated signaling events | EGFR, FYN, SRC |  | 2.18e-04 |
| Signaling events mediated by Hepatocyte Growth Factor Receptor (c-Met) | AKT1, CTNNB1, JUN, MAPK1, SRC |  | 2.26e-04 |
| Hedgehog signaling events mediated by Gli proteins | AKT1, CREBBP, HDAC1, HDAC2 |  | 3.42e-04 |
| Regulation of cytoplasmic and nuclear SMAD2/3 signaling | MAPK1, SMAD2, SMAD4 |  | 3.57e-04 |
| Class I PI3K signaling events | FYN, HSP90AA1, LYN, SRC |  | 3.70e-04 |
| FoxO family signaling | AKT1, CREBBP, CTNNB1, EP300 |  | 3.99e-04 |
| Ceramide signaling pathway | AKT1, MAPK1, MYC, RB1 |  | 3.99e-04 |
| RAC1 signaling pathway | CTNNB1, JUN, STAT3, STAT5A |  | 5.72e-04 |
| VEGFR3 signaling in lymphatic endothelium | AKT1, CREB1, MAPK1 |  | 6.97e-04 |
| Signaling events mediated by focal adhesion kinase | FYN, JUN, MAPK1, SRC |  | 9.48e-04 |
| VEGFR1 specific signals | AKT1, HSP90AA1, MAPK1 |  | 1.19e-03 |
| Ephrin B reverse signaling | FYN, LYN, SRC |  | 1.19e-03 |
| BCR signaling pathway | AKT1, JUN, LYN, MAPK1 |  | 1.25e-03 |
| Regulation of p38-alpha and p38-beta | FYN, LYN, SRC |  | 1.31e-03 |
| Nephrin/Neph1 signaling in the kidney podocyte | AKT1, FYN, JUN |  | 1.31e-03 |
| CDC42 signaling events | CTNNB1, JUN, MAPK1, SRC |  | 1.39e-03 |
| Aurora A signaling | AKT1, MDM2, TP53 |  | 1.43e-03 |
| Netrin-mediated signaling events | FYN, MAPK1, SRC |  | 1.43e-03 |
| EPHA forward signaling | FYN, LYN, SRC |  | 1.71e-03 |
| IL12 signaling mediated by STAT4 | CREBBP, JUN, STAT3 |  | 1.86e-03 |
| Class I PI3K signaling events mediated by Akt | AKT1, HSP90AA1, SRC |  | 2.01e-03 |
| HIF-2-alpha transcription factor network | CREBBP, EP300, SP1 |  | 2.01e-03 |
| Integrins in angiogenesis | AKT1, HSP90AA1, MAPK1, SRC |  | 2.06e-03 |
| Validated transcriptional targets of AP1 family members Fra1 and Fra2 | EP300, JUN, SP1 |  | 2.17e-03 |
| E-cadherin signaling in the nascent adherens junction | AKT1, CTNNB1, SRC |  | 2.71e-03 |
| Plasma membrane estrogen receptor signaling | AKT1, ESR1, SRC |  | 3.10e-03 |
| CXCR3-mediated signaling events | AKT1, MAPK1, SRC |  | 3.53e-03 |
| ErbB receptor signaling network | EGFR, HSP90AA1 |  | 5.58e-03 |
| TGF-beta receptor signaling | CTNNB1, SMAD2, SMAD4 |  | 6.50e-03 |
| Validated transcriptional targets of TAp63 isoforms | EP300, MDM2, SP1 |  | 6.50e-03 |
| Hypoxic and oxygen homeostasis regulation of HIF-1-alpha | HSP90AA1, TP53 |  | 7.79e-03 |
| SHP2 signaling | EGFR, JAK2, STAT1 |  | 7.84e-03 |
| Coregulation of Androgen receptor activity | AKT1, AR, CTNNB1 |  | 9.32e-03 |
| Signaling events mediated by PRL | MAPK1, SRC |  | 1.12e-02 |
| p75(NTR)-mediated signaling | AKT1, E2F1, TP53 |  | 1.23e-02 |
| S1P2 pathway | JUN, MAPK1 |  | 1.41e-02 |
| ALK1 signaling events | MAPK1, SMAD4 |  | 1.51e-02 |
| IL8- and CXCR1-mediated signaling events | AKT1, LYN |  | 1.73e-02 |
| Reelin signaling pathway | AKT1, FYN |  | 1.73e-02 |
| Nectin adhesion pathway | CTNNB1, SRC |  | 1.84e-02 |
| Osteopontin-mediated events | JUN, MAPK1 |  | 1.95e-02 |
| E-cadherin signaling events | CTNNB1 |  | 2.15e-02 |
| Ephrin A reverse signaling | FYN |  | 2.15e-02 |
| Syndecan-2-mediated signaling events | MAPK1, SRC |  | 2.18e-02 |
| IL8- and CXCR2-mediated signaling events | AKT1, LYN |  | 2.43e-02 |
| Trk receptor signaling mediated by the MAPK pathway | CREB1, MAPK1 |  | 2.43e-02 |
| Arf6 signaling events | EGFR, SRC |  | 2.55e-02 |
| FAS (CD95) signaling pathway | AKT1, SRC |  | 2.94e-02 |
| EPHB forward signaling | MAPK1, SRC |  | 3.07e-02 |
| Internalization of ErbB1 | EGFR, SRC |  | 3.21e-02 |
| Urokinase-type plasminogen activator (uPA) and uPAR-mediated signaling | EGFR, SRC |  | 3.35e-02 |
| BMP receptor signaling | MAPK1, SMAD4 |  | 3.48e-02 |
| Stabilization and expansion of the E-cadherin adherens junction | CTNNB1, EGFR |  | 3.63e-02 |
| a6b1 and a6b4 Integrin signaling | AKT1, EGFR |  | 3.91e-02 |
| PLK3 signaling events | TP53 |  | 4.20e-02 |
| Class IB PI3K non-lipid kinase events | MAPK1 |  | 4.20e-02 |
| Calcineurin-regulated NFAT-dependent transcription in lymphocytes | E2F1, JUN |  | 4.35e-02 |
| TCR signaling in na�ve CD8+ T cells | AKT1, FYN |  | 5.43e-02 |
| Role of Calcineurin-dependent NFAT signaling in lymphocytes | CREBBP, EP300 |  | 5.75e-02 |
| EGFR-dependent Endothelin signaling events | EGFR |  | 6.17e-02 |
| Neurotrophic factor-mediated Trk receptor signaling | MAPK1, STAT3 |  | 6.39e-02 |
| TCR signaling in na�ve CD4+ T cells | AKT1, FYN |  | 7.39e-02 |
| ALK2 signaling events | SMAD4 |  | 7.44e-02 |
| mTOR signaling pathway | AKT1, MAPK1 |  | 7.72e-02 |
| Downstream signaling in na�ve CD8+ T cells | JUN, MAPK1 |  | 7.89e-02 |
| JNK signaling in the CD4+ TCR pathway | JUN |  | 9.27e-02 |
| S1P4 pathway | MAPK1 |  | 9.27e-02 |
| Ras signaling in the CD4+ TCR pathway | MAPK1 |  | 9.27e-02 |
| Arf6 downstream pathway | MAPK1 |  | 9.86e-02 |
| LPA4-mediated signaling events | CREB1 |  | 1.04e-01 |
| Atypical NF-kappaB pathway | SRC |  | 1.10e-01 |
| Degradation of beta catenin | CTNNB1 |  | 1.16e-01 |
| EPHA2 forward signaling | SRC |  | 1.21e-01 |
| S1P1 pathway | MAPK1 |  | 1.32e-01 |
| p38 signaling mediated by MAPKAP kinases | CREB1 |  | 1.32e-01 |
| Canonical Wnt signaling pathway | CTNNB1 |  | 1.32e-01 |
| PDGFR-alpha signaling pathway | JUN |  | 1.38e-01 |
| Cellular roles of Anthrax toxin | MAPK1 |  | 1.38e-01 |
| Signaling events mediated by the Hedgehog family | AKT1 |  | 1.43e-01 |
| Alpha9 beta1 integrin signaling events | SRC |  | 1.53e-01 |
| C-MYC pathway | MYC |  | 1.53e-01 |
| RXR and RAR heterodimerization with other nuclear receptor | NCOR2 |  | 1.63e-01 |
| TRAIL signaling pathway | MAPK1 |  | 1.68e-01 |
| Insulin-mediated glucose transport | AKT1 |  | 1.73e-01 |
| BARD1 signaling events | TP53 |  | 1.77e-01 |
| Calcium signaling in the CD4+ TCR pathway | JUN |  | 1.82e-01 |
| IGF1 pathway | AKT1 |  | 1.82e-01 |
| Alpha4 beta1 integrin signaling events | SRC |  | 1.95e-01 |
| ATM pathway | MDM2 |  | 1.95e-01 |
| N-cadherin signaling events | CTNNB1 |  | 2.04e-01 |
| IL1-mediated signaling events | JUN |  | 2.04e-01 |
| ATR signaling pathway | MDM2 |  | 2.20e-01 |
| Insulin Pathway | AKT1 |  | 2.39e-01 |
| RhoA signaling pathway | JUN |  | 2.42e-01 |
| TNF receptor signaling pathway | STAT1 |  | 2.49e-01 |
| Validated transcriptional targets of deltaNp63 isoforms | MDM2 |  | 2.49e-01 |
| Syndecan-1-mediated signaling events | MAPK1 |  | 2.53e-01 |
| Arf6 trafficking events | CTNNB1 |  | 2.53e-01 |
