# Supplementary material for: A simple knowledge-based mining method for exploring hidden key molecules in a human biomolecular network
Source: BMC Syst Biol. 2012 Sep 15;6:124. doi: 10.1186/1752-0509-6-124 (PMC3740779; doi:10.1186/1752-0509-6-124)
Supplement: Additional file 2 — The collection of results for the Pathway Interaction Database analysis. The index.html file contains the links to the Pathway Interaction Database results for the various input genes. The input genes consist of the results of NetHiKe and Hubba (the top 30 genes of each). (Mini-websites, browse the index.html. [file 1752-0509-6-124-S2.zip › mini_web/index.html]

Additional File 2


# Additional File 2 (The Pathway Interaction Database results)

A: NetHiKe  
B: Hubba (degree)  
C: Hubba (bottle neck)  
D: Hubba (EPC)  
E: Hubba (DMNC)  
F: Hubba (betweenness centrality)  
G: NetHiKe(NRG2 weight x2.0)  
H: NetHiKe(NRG2 weitht x20.0)  
