# Supplementary figures and images for: A simple knowledge-based mining method for exploring hidden key molecules in a human biomolecular network
Source: BMC Syst Biol. 2012 Sep 15;6:124. doi: 10.1186/1752-0509-6-124 (PMC3740779; doi:10.1186/1752-0509-6-124)

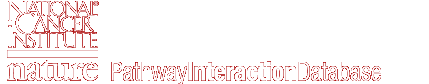

Supplement: Additional file 2 — The collection of results for the Pathway Interaction Database analysis. The index.html file contains the links to the Pathway Interaction Database results for the various input genes. The input genes consist of the results of NetHiKe and Hubba (the top 30 genes of each). (Mini-websites, browse the index.html. [file 1752-0509-6-124-S2.zip › mini_web/src/journal_header.gif]

Supplement: Additional file 2 — The collection of results for the Pathway Interaction Database analysis. The index.html file contains the links to the Pathway Interaction Database results for the various input genes. The input genes consist of the results of NetHiKe and Hubba (the top 30 genes of each). (Mini-websites, browse the index.html. [file 1752-0509-6-124-S2.zip › mini_web/src/skipnav.gif]

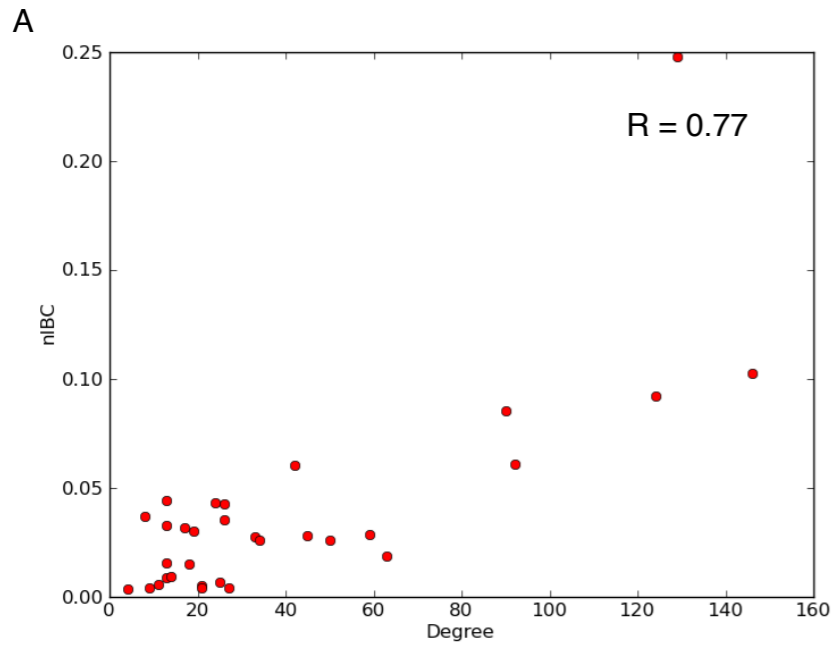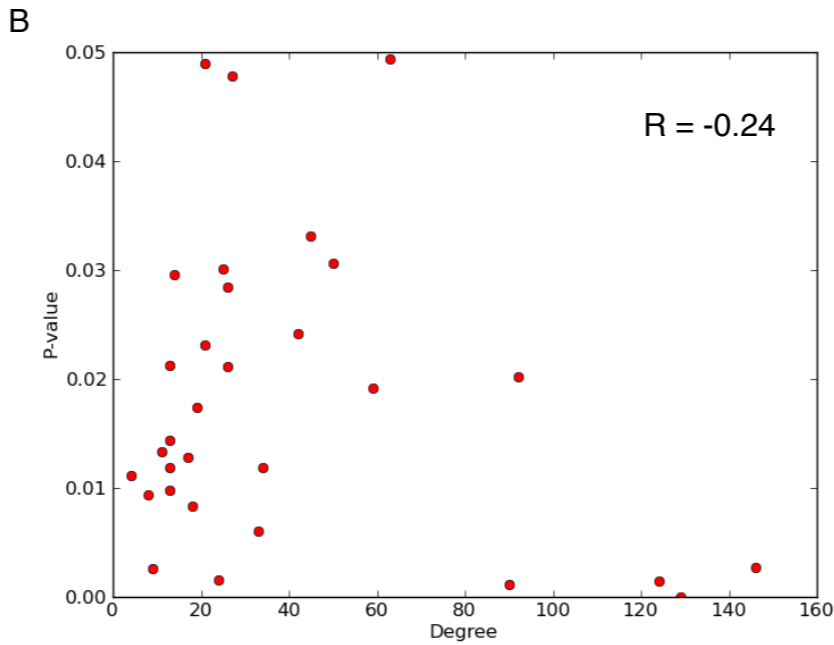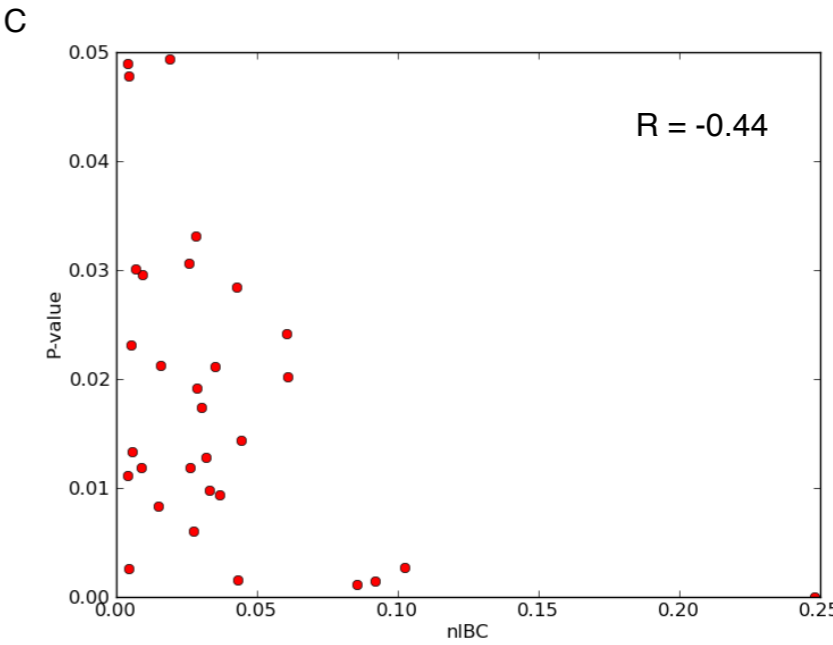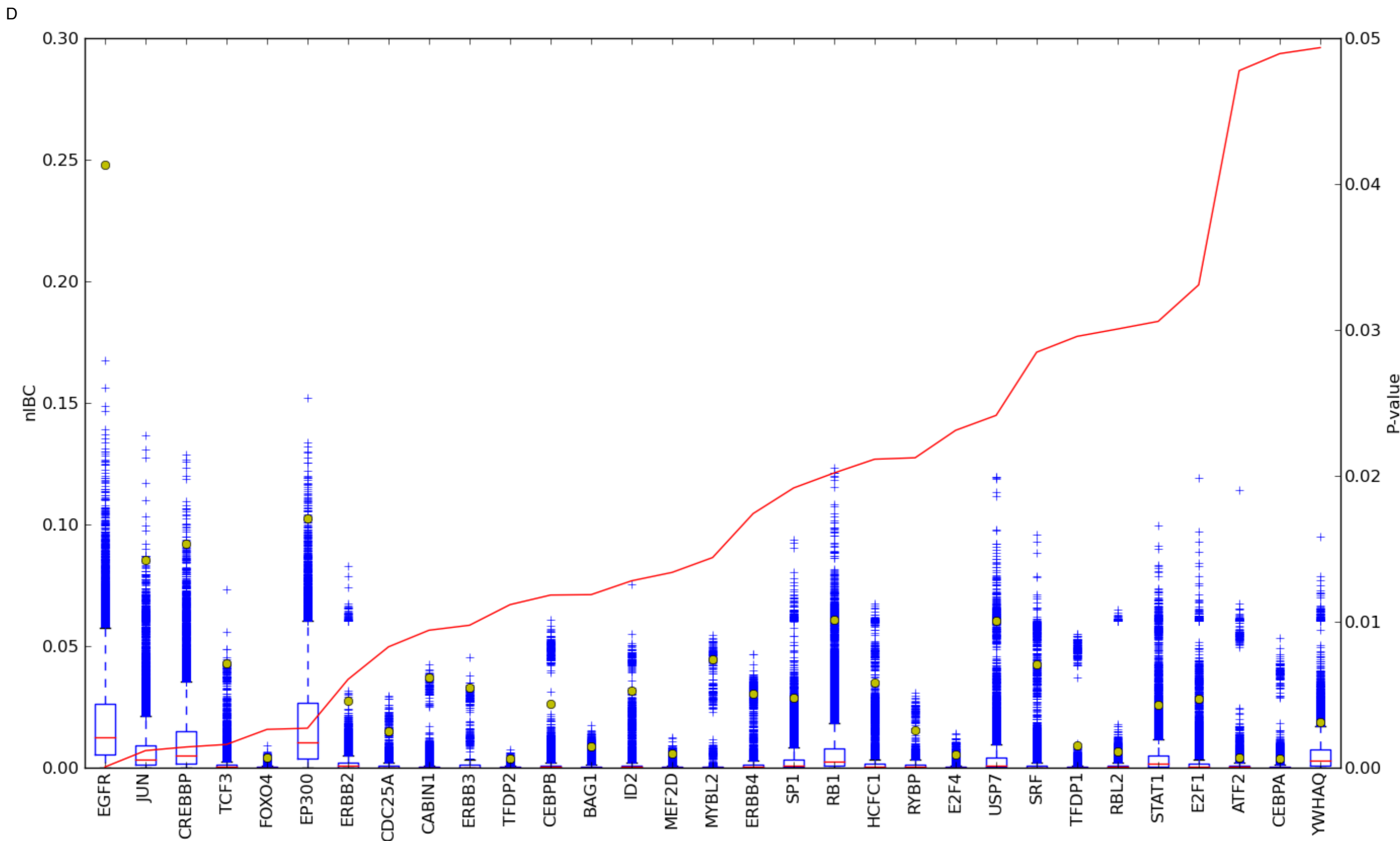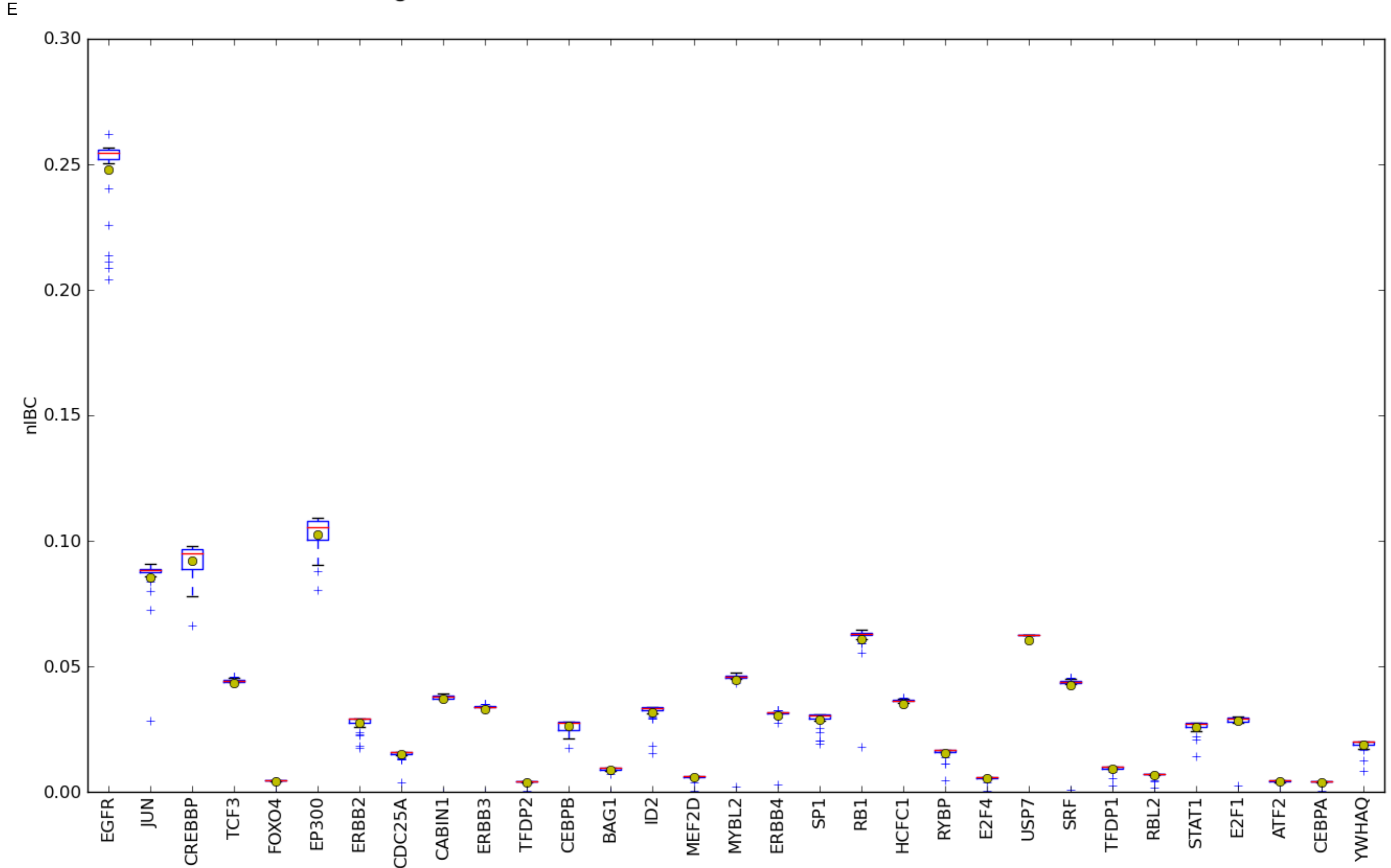

Supplement: Additional file 3 — Degree, P-value, and nlBC for the ErbB pathway analysis data. Plots of the degrees, p-values, and nlBC values of genes with P<0.05 in the results of the ErbB pathway analysis (A-C) and boxplots of the nlBC values (D and E). A) Plot of the node degrees in the background network vs. nlBC. B) Degree vs. simulated p-values. C) nlBC vs. p-values. D) Boxplot visualization of the genes in Table 1. The boxes are the nlBC values generated from randomly selected genes to calculate the simulated p-values, and the yellow dots denotes the actual nlBC value that was calculated based on the input genes (listed in the Additional file 1). The simulated p-values, listed in the Table 1, are plotted as the red line associated with the right axis. E) The nlBC values that were generated by a leave-one-out method using the input genes, and the actual nlBC values as the yellow dots. The plot D and E have the same Y-axis scale (left) and the gene order in X-axis. [file 1752-0509-6-124-S3.pdf]

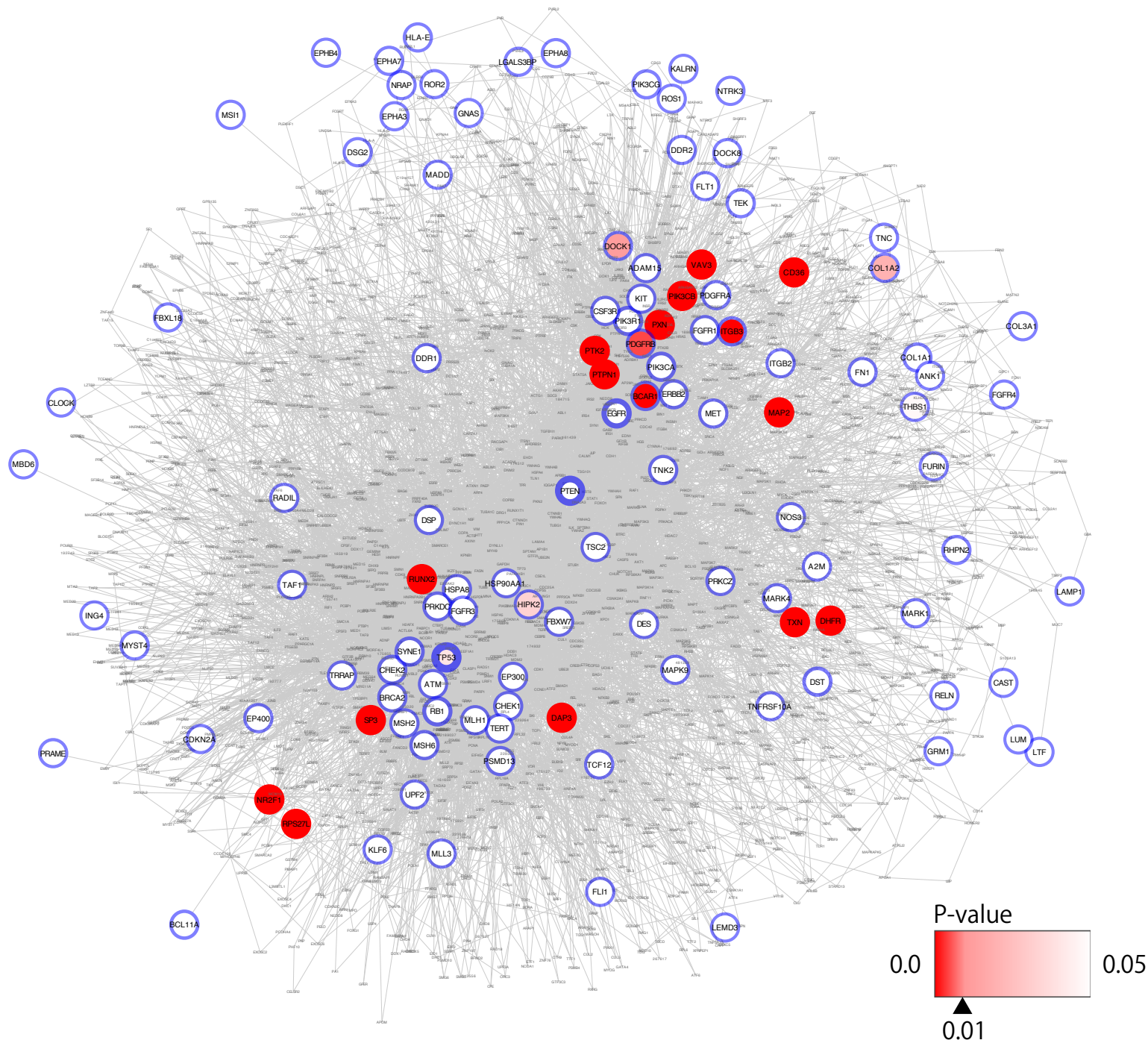

Supplement: Additional file 6 — The GBM network and key molecules inferred by NetHiKe. The extracted network made by genes mutated in GBM. The blue-bordered nodes are the input nodes, and the p-values are shown by the depth of the red color. [file 1752-0509-6-124-S6.pdf]

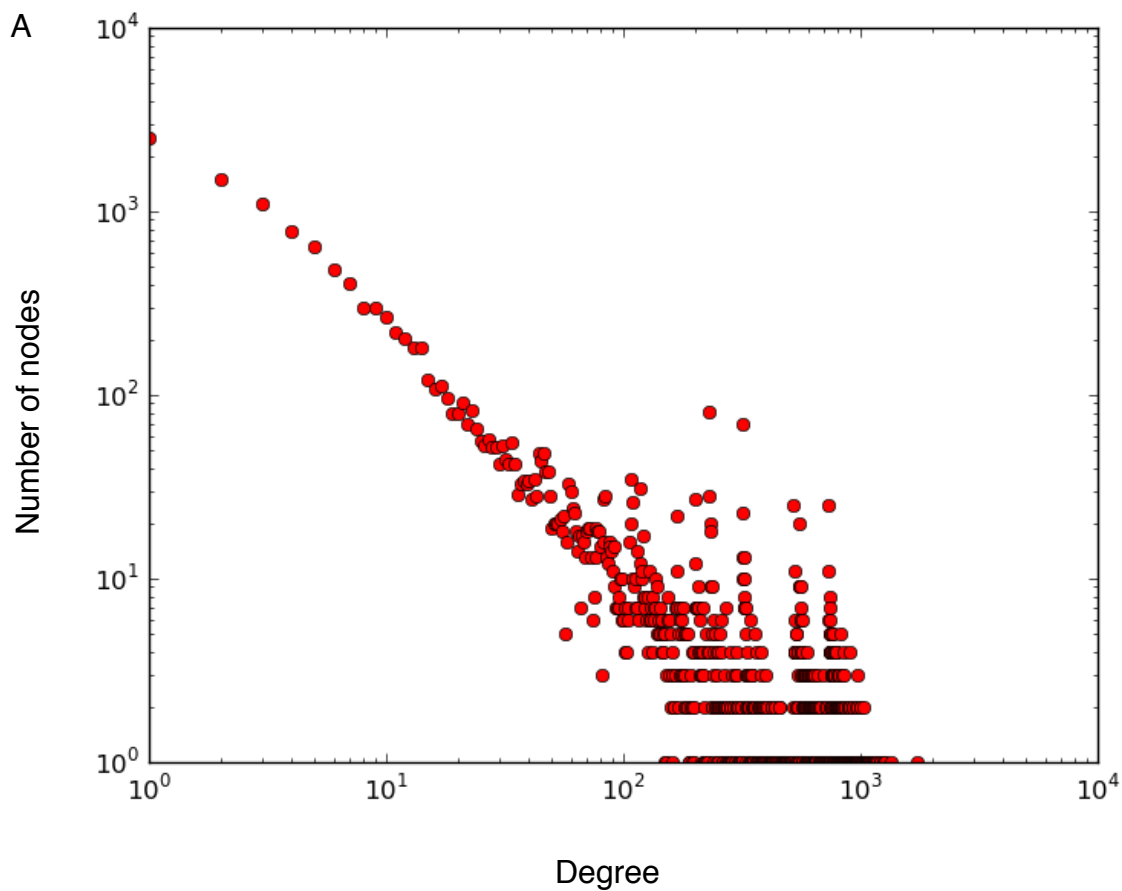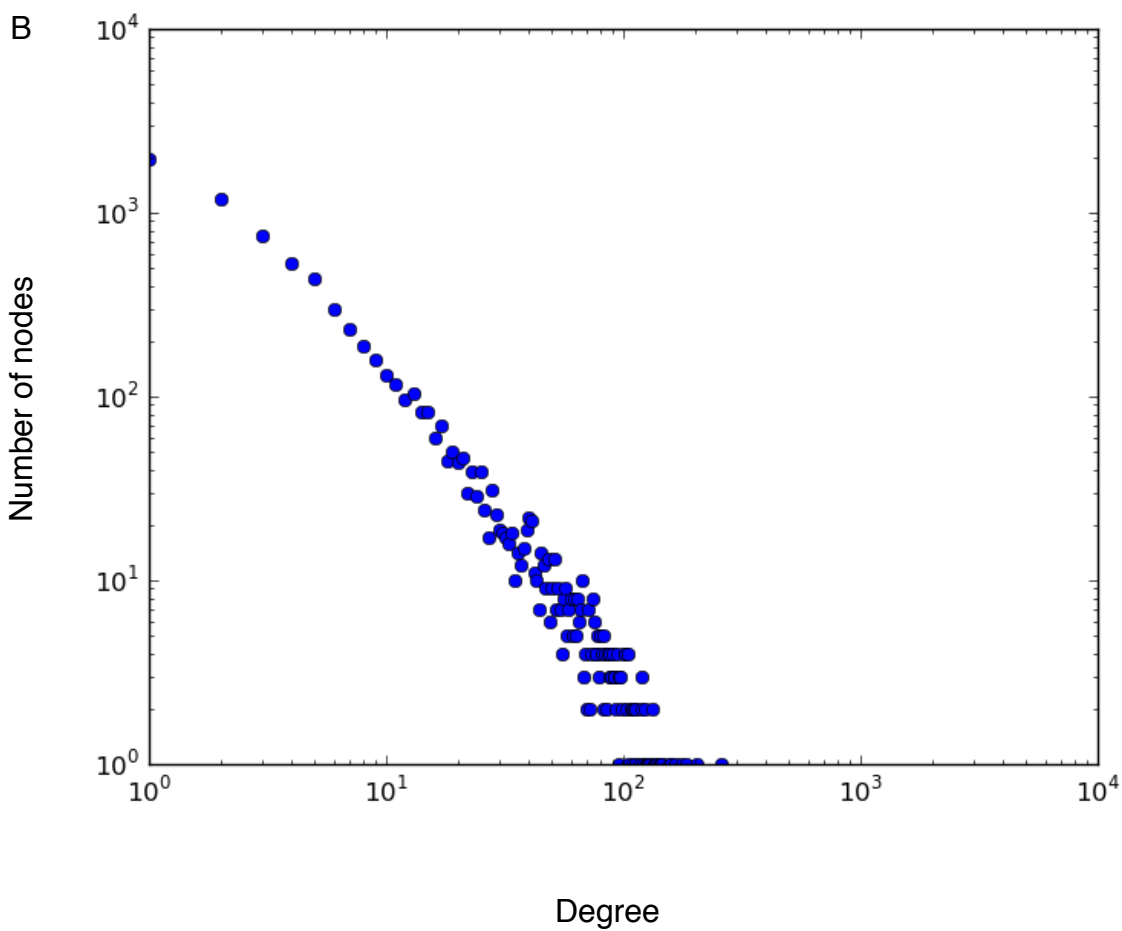

Supplement: Additional file 8 — Two different degree distributions depend on the edge selection. Log-log degree distribution for the network constructed from the whole Pathway Commons data (A) and the selected edges (B). [file 1752-0509-6-124-S8.pdf]
