# Supplementary material for: A simple knowledge-based mining method for exploring hidden key molecules in a human biomolecular network
Source: BMC Syst Biol. 2012 Sep 15;6:124. doi: 10.1186/1752-0509-6-124 (PMC3740779; doi:10.1186/1752-0509-6-124)
Supplement: Additional file 4 — Comparison lists of the top 30 genes on NetHiKe and Hubba. The lists of the top 30 genes generated by NetHiKe and Hubba with the same input data. In the Hubba analysis, six different methods were used. The six-digit number indicates the Pathway Commons ID, as the molecules do not have general gene names. [file 1752-0509-6-124-S4.pdf]

			Hubba results											
rank	NetHike	degree	Degree	degree	BottleNeck	degree	EPC	degree	MNC	degree	DMNC	degree	Betweenness	degree
1	EGFR	129	CREBBP	124	ESR1	120	ESR1	120	ESR1	120	ERBB4	19	EGF	16
2	JUN	90	ESR1	120	TP53	132	JUN	90	CREBBP	124	STAT6	12	STAT1	50
3	CREBBP	124	EGFR	129	CREBBP	124	EP300	146	EP300	146	E2F5	5	CDK1	79
4	TCF3	24	TP53	132	USP7	42	CREBBP	124	JUN	90	FOS	8	JAK2	73
5	FOXO4	9	JUN	90	EGFR	129	RB1	92	RB1	92	PTPN11	82	175868	257
6	EP300	146	RB1	92	161596	205	AR	132	STAT3	67	VDR	17	ZBTB16	65
7	ERBB2	33	EP300	146	SMAD4	97	STAT3	67	AR	132	CTNNB1	119	VIM	101
8	CDC25A	18	SMAD4	97	EP300	146	TP53	132	SRC	134	PTPN1	33	EGFR	129
9	CABIN1	8	STAT3	67	CDKN1A	47	SMAD4	97	SMAD4	97	FOXO4	9	MAPK1	71
10	ERBB3	13	SRC	134	JUN	90	EGFR	129	EGFR	129	NRIP1	25	RB1	92
11	TFDP2	4	AR	132	SP1	59	SP1	59	NR3C1	60	NCOA1	48	ERBB4	19
12	CEBPB	34	STAT1	50	SRC	134	SRC	134	TP53	132	TBP	81	EP300	146
13	BAG1	13	CREB1	43	CREB1	43	HDAC1	117	E2F1	45	EGF	16	STAT3	67
14	ID2	17	E2F1	45	TRAF2	142	NR3C1	60	HDAC1	117	NCOA2	30	HSP90AA1	166
15	MEF2D	11	SP1	59	PIK3R1	102	MDM2	62	SP1	59	STAT4	7	HDAC1	117
16	MYBL2	13	USP7	42	RB1	92	STAT1	50	JAK2	73	STAT2	9	E2F4	21
17	ERBB4	19	NR3C1	60	STAT1	50	MYC	82	STAT1	50	RBL2	25	UBQLN4	125
18	SP1	59	161596	205	AR	132	SMAD2	102	MYC	82	SKP2	29	TRAF2	142
19	RB1	92	MAPK1	71	TEX11	23	CEBPB	34	MDM2	62	PELP1	23	AR	132
20	HCFC1	26	MDM2	62	NR3C1	60	HSP90AA1	166	MAPK1	71	174932	28	CREB1	43
21	RYBP	13	HDAC1	117	E2F1	45	E2F1	45	SMAD2	102	CDK2	51	JUN	90
22	E2F4	21	ZBTB16	65	UBQLN4	125	AKT1	85	HDAC2	105	KAT2B	43	CREBBP	124
23	USP7	42	JAK2	73	HCFC1	26	HDAC2	105	CEBPB	34	ERBB3	13	SMAD4	97
24	SRF	26	HDAC2	105	HSP90AA1	166	MAPK1	71	AKT1	85	RELA	94	SRC	134
25	TFDP1	14	PIK3R1	102	RING1	37	NCOR2	89	161596	205	E2F4	21	161596	205
26	RBL2	25	YY1	45	STAT3	67	161596	205	YY1	45	STAT5A	24	E2F1	45
27	STAT1	50	SMAD2	102	ERBB4	19	CREB1	43	STAT5A	24	CEBPA	21	TP53	132
28	E2F1	45	SIN3A	89	VIM	101	JAK2	73	PIK3R1	102	PTPN6	66	USP7	42
29	ATF2	27	HSP90AA1	166	BMI1	25	CTNNB1	119	JAK1	53	DNM1	35	SP1	59
30	CEBPA	21	MYC	82	TCF3	24	HDAC4	41	HSP90AA1	166	SP1	59	ESR1	120
		38.87		94.87		86.80		95.70		93.43		35.07		98.63

The number of six-digit means the Pathway Commons ID. The molecules don't have gene names.
